# Differing House Finch Cytokine Expression Responses to Original and Evolved Isolates of *Mycoplasma gallisepticum*

**DOI:** 10.3389/fimmu.2018.00013

**Published:** 2018-01-22

**Authors:** Michal Vinkler, Ariel E. Leon, Laila Kirkpatrick, Rami A. Dalloul, Dana M. Hawley

**Affiliations:** ^1^Faculty of Science, Department of Zoology, Charles University, Prague, Czechia; ^2^Department of Biological Sciences, Virginia Tech, Blacksburg, VA, United States; ^3^Avian Immunobiology Laboratory, Department of Animal and Poultry Sciences, Virginia Tech, Blacksburg, VA, United States

**Keywords:** avian pathogen, bird cytokine signalling, disease ecology, emerging infectious diseases, evolution of virulence, host–parasite interaction, periocular inflammation, wild immunology

## Abstract

The recent emergence of the poultry bacterial pathogen *Mycoplasma gallisepticum* (MG) in free-living house finches (*Haemorhous mexicanus*), which causes mycoplasmal conjunctivitis in this passerine bird species, resulted in a rapid coevolutionary arms-race between MG and its novel avian host. Despite extensive research on the ecological and evolutionary dynamics of this host–pathogen system over the past two decades, the immunological responses of house finches to MG infection remain poorly understood. We developed seven new probe-based one-step quantitative reverse transcription polymerase chain reaction assays to investigate mRNA expression of house finch cytokine genes (*IL1B, IL6, IL10, IL18, TGFB2, TNFSF15*, and *CXCLi2*, syn. *IL8L*). These assays were then used to describe cytokine transcription profiles in a panel of 15 house finch tissues collected at three distinct time points during MG infection. Based on initial screening that indicated strong pro-inflammatory cytokine expression during MG infection at the periorbital sites in particular, we selected two key house finch tissues for further characterization: the nictitating membrane, i.e., the internal eyelid in direct contact with MG, and the Harderian gland, the secondary lymphoid tissue responsible for regulation of periorbital immunity. We characterized cytokine responses in these two tissues for 60 house finches experimentally inoculated either with media alone (sham) or one of two MG isolates: the earliest known pathogen isolate from house finches (VA1994) or an evolutionarily more derived isolate collected in 2006 (NC2006), which is known to be more virulent. We show that the more derived and virulent isolate NC2006, relative to VA1994, triggers stronger local inflammatory cytokine signaling, with peak cytokine expression generally occurring 3–6 days following MG inoculation. We also found that the extent of pro-inflammatory interleukin 1 beta signaling was correlated with conjunctival MG loads and the extent of clinical signs of conjunctivitis, the main pathological effect of MG in house finches. These results suggest that the pathogenicity caused by MG infection in house finches is largely mediated by host pro-inflammatory immune responses, with important implications for the dynamics of host–pathogen coevolution.

## Introduction

Emerging infectious diseases apply novel and powerful selection pressures on wildlife host immune responses. There is a growing list of examples of wildlife hosts that have rapidly evolved resistance or tolerance to recently emerged infectious diseases [e.g., rabbits and myxoma virus (1); amphibians and chytridiomycosis (2); bats and white-nose syndrome (3); and house finches and mycoplasmal conjunctivitis (4)]. However, due to a historical dearth of techniques available for characterizing immune responses in non-model systems (5), we still know relatively little about the immune responses of natural wildlife hosts to well-studied emerging diseases, particularly for non-mammalian hosts (6, 7). Furthermore, there have been few opportunities to experimentally characterize how pathogen evolution following initial disease emergence has altered the immune responses of natural wildlife hosts to infection (8, 9).

Here, we use a well-studied and recently emerged wildlife disease system—the bacterium *Mycoplasma gallisepticum* (MG) and its novel songbird host, the house finch—to characterize cytokine responses during *in vivo* infection, and to understand how cytokine responses differ for birds inoculated with an original versus evolved pathogen isolate. MG is an economically significant pathogen of poultry, where it largely causes a chronic respiratory disease (10). In the early to mid-1990s, a novel clade of this pathogen emerged in house finches (11), causing severe conjunctivitis (12), and resulting in significant decreases (up to 60%) in the size of free-living house finch populations (13). Since its initial detection in 1994, MG spread rapidly across the United States (14) and evolved both genotypically (15, 16) and phenotypically (17), with rapid increases in virulence and pathogenicity observed following MG’s establishment on each coast of the United States (18). Thus, while original isolates (e.g., VA1994) produce moderately severe but often self-healing disease in captivity, evolutionarily derived isolates (e.g., NC2006) are more likely to cause severe and/or chronic infection and disease (9, 19).

The house finch–MG interaction has become an important natural model of coevolution between a host and an emerging pathogen, facilitating insights into fundamental issues in disease ecology and evolutionary biology (18, 20, 21). However, we still have only a limited understanding of the key immunological features of the house finch response to MG. Several studies show that house finch immune responses to MG are associated with hematological changes (22, 23) and antigen-specific antibody production in both lachrymal fluid and blood (9, 24). Nevertheless, the protective effects of humoral immunity remain unclear (19). Studies of house finch gene expression in the spleen using suppression subtractive hybridization and cDNA microarrays identified several immune response genes differentially expressed 14 days after experimental inoculation with MG (25), with upregulation of many immune response genes in birds from a resistant population relative to a susceptible population (4). Differential expression of immunity genes was also detected in house finch spleens on day 3 postinoculation (26), with population differences at that time point suggesting that differences in innate immunity might be important for host resistance. However, the role of cytokines, shown to be upregulated early in poultry infection with MG at the infection site (27), could not be elucidated in this study. Using the ratio of interleukin (IL)-1β (*IL1B*) to *IL10* mRNA expression in whole blood 24 h after experimental inoculation with MG, Adelman et al. (28) provided evidence for a potential association between early inflammatory cytokine responses and the degree of inflammation caused by a given load of MG. Thus, inflammatory immune responses may play an important role in host defense to this disease, but these responses have not yet been well examined during MG infection in house finches. Furthermore, despite the existence of archived MG isolates that span the course of this emerging disease (29), no studies to date have examined how inflammatory immune responses differ for hosts inoculated with an early, less virulent isolate of MG versus a more derived, virulent isolate (18).

Inflammation is one of the most important mechanisms of pathogen clearance in vertebrate immunity (30). As a self-damaging immunological process, inflammation is carefully regulated by highly coordinated cytokine signaling. In birds, pro-inflammatory cytokines such as IL1B, IL6, IL18, tumor necrosis factor superfamily members (TNFSF), and various chemokines (e.g., IL8 homologs including avian CXCLi2), together with anti-inflammatory cytokines including IL10 and transforming growth factor-β (TGFB), guide the immune response to avoid severe host damage associated with immunopathology (31, 32). Initial variation in the balance between these cytokines in different tissues may be responsible for the difference between successful and unsuccessful pathogen elimination. Here, using quantitative reverse transcription polymerase chain reaction (RT-qPCR), we investigate the temporal dynamics and mechanisms of MG-induced inflammation in the house finch. Furthermore, we examine whether differences in cytokine signaling are linked to the higher virulence associated with more recently evolved isolates of MG (18). To provide a comprehensive view, we described the house finch immune response to MG in a panel of 15 house finch tissues. We then examined a subset of tissues that showed strongest responses to MG infection at three time points over the course of MG infection with two distinct MG isolates. We identify differences between an original and evolutionary-derived MG isolate in their capacity to trigger expression of key inflammatory cytokine genes (*IL1B, IL6, IL10, IL18, TGFB2, CXCLi2*, and *TNFSF15*) leading to signaling that may be crucial for the development of MG-induced immunopathology.

## Materials and Methods

### Animals

Sixty house finches of mixed sex (34 males, 26 females) were trapped in June–July 2015 (31 hatch-year individuals) and December 2015 (29 individuals of unknown age) *via* mist net or cage traps placed around feeders at sites located in Montgomery County, VA, USA or within the city of Radford, VA, USA (all capture sites were within 25 km of each other). House finches can only be accurately aged by plumage before completion of the pre-basic molt in September–October, and thus birds captured in December were of unknown age. Immediately following capture, birds were housed individually or in pairs in wire-mesh cages (76 cm × 46 cm × 46 cm) in a Biosafety Level 1 animal facility with constant daylength (12L:12D) and temperature (21–22°C) and provided with drinking water and pelleted diet *ad libitum* (Daily Maintenance Diet, Roudybush Inc., Woodland, CA, USA). All birds underwent a 2-week quarantine following capture to ensure they had no exposure to MG before capture. In brief, birds were captured and assessed every 3–4 days for the presence of visible eye lesions (see methods below). On day 14 following capture (to account for time for development of antibodies if exposure to MG occurred on the day of capture), all birds were tested for MG-specific antibodies using an Idexx FlockCheck MG antibody ELISA kit (IDEXX, Westbrook, ME, USA) with modifications described in Ref. (33). Only individuals that were seronegative, never showed clinical signs, and were never housed with a cagemate that showed clinical signs were used in this experiment. Because their immune systems were still maturing at the time of capture, animals caught in July–August were treated preventatively with Cankarex and sulfadimethoxine (see Supplementary Material) in their drinking water to prevent overgrowth of *Trichomonas* and coccidial parasites, respectively. Following quarantine, mass and tarsus length (estimate of size) were measured in all birds used in this experiment, and the birds were moved to individual cages, but all other housing conditions remained unchanged. Animal capture was approved by federal (USFWS permit MB158404-1) and state (VDGIF permit 050352) agencies, and procedures for animal care and use were approved by Virginia Tech’s Institutional Animal Care and Use Committees.

### MG Isolates

Expansions of MG field isolates were acquired from the Mycoplasma Diagnostic and Research Laboratory at the NC State University College of Veterinary Medicine [ADRL NCSU CVM (29)]. For our study, we selected two MG isolates: (1) isolate VA1994 that is an expansion of the earliest collected MG isolate from a free-living house finch with conjunctivitis, collected in Virginia in 1994 shortly after MG first emerged in house finches [(34); ADRL NCSU CVM Accession No. 7994-1 (7P) 2/12/09] and (2) isolate NC2006 that is an expansion of a more evolutionarily derived isolate collected from a house finch in North Carolina with conjunctivitis in 2006 [ADRL NCSU CVM Accession No. 2006.080-5-4P 7/26/12]. Upon thawing, isolates were diluted in Frey’s media with 15% swine serum (FMS) to match the suspension concentration of 2 × 10^4^ color changing units/mL.

### Experimental Design and Timeline

To quantify house finch cytokine responses to an original and more evolved isolate of MG, we used three treatment groups (Table 1): (1) sham-inoculated controls (*n* = 12), (2) NC2006-inoculated experimental group (*n* = 24), and (3) VA1994-inoculated experimental group (*n* = 24). To capture the temporal dynamics of cytokine expression, birds were further divided within each group into three equally sized time-point batches (*n* = 4 per time point in the case of controls and *n* = 8 per time point in the case of experimental groups). All groups were designed to contain approximately equal proportions of males and females, and hatch-year birds and individuals of unknown age (Table S1 in Supplementary Material), but otherwise assignments to treatments were random. We tested for pretreatment differences in size or mass and no significant differences in these traits were present (all *P* > 0.05).

**Table 1:** Design of *Mycoplasma gallisepticum* (MG) inoculation experiment.

Treatment	Day 3 PI	Day 6 PI	Day 13 PI
Control	*N* = 4	*N* = 4	*N* = 4
VA1994	*N* = 8	*N* = 8	*N* = 8
NC2006	*N* = 8	*N* = 8	*N* = 8

*House finches were inoculated with sham treatment (media alone) or one of two MG isolates (VA1994 or NC2006) on day 0. Equal sample sizes from each treatment group were euthanized on one of each of the three post inoculation (PI) time points*.

The experiment was conducted in January–February 2016. On day 0, the mass of all individuals was measured, and conjunctiva was swabbed for qPCR to ensure that no birds harbored baseline loads of MG (see below for details). Thereafter, all birds were treated with the respective inoculum: experimental birds were inoculated with 35 µL of MG suspended in FMS (either isolate NC2006 or VA1994) administered directly into the palpebral conjunctiva of each eye *via* micropipette (70 µL in total), while sham control individuals were given the same volume of FMS alone. Afterward, birds were held in individual paper lunchbags for approximately 5 min to allow full absorption of inoculum, and then released back into their home cages and left undisturbed. On day 3 post inoculation (DPI 3), birds assigned to the first time point batch were eye scored (see below), conjunctival swabs were collected (see below), and the birds were euthanized *via* rapid decapitation to collect fresh tissue samples and prevent confounding effects of chemical inhalants on gene expression. The same procedure was performed with birds assigned to the second time point batch on DPI 6 and to the third time point batch on DPI 13. All manipulation with experimental animals (including euthanasia) was performed in the quickest and most humane way possible to minimize pain and distress. Latex gloves were changed between manipulations with each individual to prevent any inadvertent MG transmission. Because MG requires direct contact between individuals or contact with a highly contaminated surface (35), this measure is sufficient to prevent transmission between experimental birds.

### Eye Lesion Scoring and Conjunctival Swabbing

Eye lesions characteristic of mycoplasmal conjunctivitis were visually scored on a 0–3 scale as previously described (36). Briefly, no visible clinical signs were scored as 0, minor swelling and discoloration around the eye was scored as 1, moderate swelling with occasional conjunctival eversion was scored as 2, and moderate to severe swelling, conjunctival eversion, and noticeable exudate was scored as 3. Scores from each eye were combined within time points to give a total eye score ranging from 0 to 6 for each individual.

To quantify MG load, conjunctivae were gently swabbed for 5 s with a sterile cotton swab pre-dipped in sterile tryptose phosphate broth (TPB). Swabs were swirled in 300 µL of sterile TPB and then wrung out into the sample collection tube. Samples from both eyes were pooled within sampling date for a given individual and frozen at −20°C until further processing. Genomic DNA was extracted with Qiagen DNeasy 96 Blood and Tissue kits (Qiagen, Valencia, CA, USA). Extracted DNA was used to measure overall numbers of MG in the conjunctivae using a qPCR assay targeting the *mgc2* gene of MG using primers and a probe previously described (37) and qPCR methods previously outlined (18).

### Samples Used for Gene Expression Analysis

Immediately after decapitation, blood from the disconnected carotids was collected from each bird into a microcentrifuge tube containing RLT lysis buffer (Qiagen, Cat No. 79216). Then, the following tissues were collected separately into RNA*later* (Ambion, cat. No. AM7021): conjunctiva (lower external eyelid), nictitating membrane (internal eyelid), Harderian gland (HG), upper respiratory tract and choana, brain, bone marrow, liver, spleen, trachea, lungs, kidney, pancreas, duodenum, and ileum (small intestine *ca*. 1 cm proximal from the caeca). Dissection and sample collection were performed simultaneously by three persons, and all tissues were collected within 15 min after euthanasia. The collected tissue samples were stored at −80°C until total RNA extraction. RNA extraction was performed using High Pure RNA Tissue Kit v. 09 (Roche, Cat. No. 12033674001) according to the manufacturer’s instructions. The total RNA concentration in each extracted sample was then measured using NanoDrop 2000 (Thermo Scientific): range 2.7–1,335.8 ng/µL, average 197.6 ng/µL. We checked for the quality of the RNA extracted from different tissues in different batches using TapeStation (Agilent) as a service of the Genomics Research Laboratory, Biocomplexity Institute, Virginia Tech. In most tissue samples, the RNA quality was good [RNA integrity number (RIN) generally ranged between 8.4 and 9.5]. RIN was <8.0 in some lung, liver, and tracheal samples, and these tissues were thus excluded from further analysis.

### RT-qPCR Assays for Assessment of Target and Reference Gene Expression

In this study, we focused on a set of 7 selected pro-inflammatory and anti-inflammatory cytokine genes: *IL1B, IL6, IL10, IL18, TGFB2, CXCLi2* (*IL8L*), and *TNFSF15*. Pro-inflammatory *IL1B* and anti-inflammatory *IL10* were selected as key target genes based on the results of previous studies (28, 38). To be able to normalize the RT-qPCR data, we had to select an appropriate reference (house-keeping) gene to serve as an endogenous control in the analysis. We tested three reference genes: beta-actin (*ACTB*), glyceraldehyde 3-phosphate dehydrogenase (*GAPDH*), and *28S rRNA*.

Because allelic variation in primer/probe-annealing regions would importantly bias the accuracy of our RT-qPCR data, we first evaluated the sequence variation in the genes of interest in a sample of six individuals (three control and three MG infected) with spleen and liver RNA-seq data available (sequences obtained through Illumina HiSeq 2500) to check for common SNPs. The reads were filtered for hits to the genes of interest using blast with Atlantic canary (*Serinus canaria*) sequences (XM_009086347.1, XM_009091807.1, XM_009093631.1, XM_009096394.1, XM_009097367.1, XM_009097655.1, XM_009098024.1, XM_009098521.1, and XM_009102634.1) as references. The filtered reads were then quality trimmed and mapped to reference using Geneious v. 9.1.8-implemented tools. While this approach yielded good data on sequence variation for the reference genes (full-length mean coverage 600–7,000, 8,000–150,000 reads per individual and tissue), the sequence data were scarce for several cytokine genes (full-length mean coverage 0–35, 0–850 reads per individual and tissue). Therefore, to improve our information on the sequence variation in our genes of interest, we Sanger-sequenced the partial coding regions in mRNA of the cytokines in seven additional individuals and of *ACTB* and *GAPDH* in two additional individuals (sequences uploaded to NCBI GenBank under accession numbers MG587727–MG587771; for description of the PCR protocol see Table S2 in Supplementary Material).

Based on the sequence information on the genes of interest in the canary and the house finch, including the common house finch sequence variation, we designed the primers and probes for RT-qPCR that were located across exon–exon borders, avoided any interspecifically and intraspecifically variable positions and selecting primers that shared basic features for the RT-qPCR with annealing temperature standardized to 60°C (see the list in Table S3 in Supplementary Material). PCR with these primers was tested using cDNA (electrophoresis and Sanger-sequencing of amplicons was performed for assay specificity verification). Next, qPCR with synthesized standard DNA sequences (IDT, gBlocks Gene Fragments; Table S4 in Supplementary Material) was done using the iTaq™ Universal Probes One-Step Kit (BioRad, Cat. No. 172-5140) with cycling conditions following manufacturer’s instructions. Once the assay efficiency was estimated, all further RT-qPCR were performed with the same kit (iTaq™ Universal Probes One-Step Kit) and set up: final primer concentration 0.6 µM, final probe concentration 0.125 µM and mixed RNA template diluted in carrier tRNA (Qiagen, Cat. No. 1068337) enriched molecular water 1:5 (or 1:500 for *28S rRNA* qPCR); cycling conditions (1) 50°C 10 min, (2) 95°C 3 min, (3) (95°C 15 s, 60°C 60 s) × 40. All assays were performed with a template-free negative control and gBlock positive controls (Table S4 in Supplementary Material) in a freshly prepared dilution series. Calibrator samples were included in all assays to check for interplate RT-qPCR variation. Based on the results of GeNorm and RefFinder analyses (39, 40), *28S rRNA* was selected as a reference gene for the RT-qPCR assays (see Supplementary Material).

### RT-qPCR Data Analysis

Before data analysis, any technical replicates with Cq values highly deviating from the other two measurements in the triplicate (difference greater than 1.5, indicating error in PCR) were excluded from the calculation of Cq means. To select candidate tissues of interest, we first performed an initial screening of the qPCR data on *IL1B* and *TGFB2* across all 15 tissues in two controls and two NC2006-infected individuals at DPI 6 by using the relative quantification method described by Pfaffl (41). Here, the relative expression ratio (*R*) is calculated as *R* = (*E*_T_)Δ^Cq^*^T^*/(*E*_R_)Δ^Cq^*^R^*, where *E*_T_ is the mean amplification efficiency of the particular assay for a target gene (cytokine), *E*_R_ is the mean amplification efficiency of the particular assay for a reference gene (*28S rRNA*), the ΔCq*T* is the difference in Cq values between control mean and the treatment mean in the target gene (cytokine), and ΔCq*R* is the difference in Cq values between control mean and the treatment mean in the reference gene (*28S rRNA*). Then, for the purpose of statistical testing of the differences in cytokine expression between controls and treatments in the selected tissues, we calculated the relative RNA quantities (*Q*) as *Q* = *E*Δ^Cq^, where *E* is the mean amplification efficiency of the particular assay and ΔCq is the difference between an arbitrary Cq value chosen for the gene (in our case the lowest Cq value in the data set) and the sample Cq (42, 43). The level of *28S rRNA* expression was used as a normalization factor to standardize RNA quantities for each target gene, providing standardized expression quantity, stQ = *Q*_TARGET_/*Q_28S rRNA_*. Absolute quantity (aQ) was calculated as number of target gene copies per nanogram of the total extracted RNA. Target gene copy number was estimated based on the average standard curve equation obtained from a dilution series of a calibrated synthetic standard (gBlock sequences of known DNA copy number in the solution).

**Figure 1:**
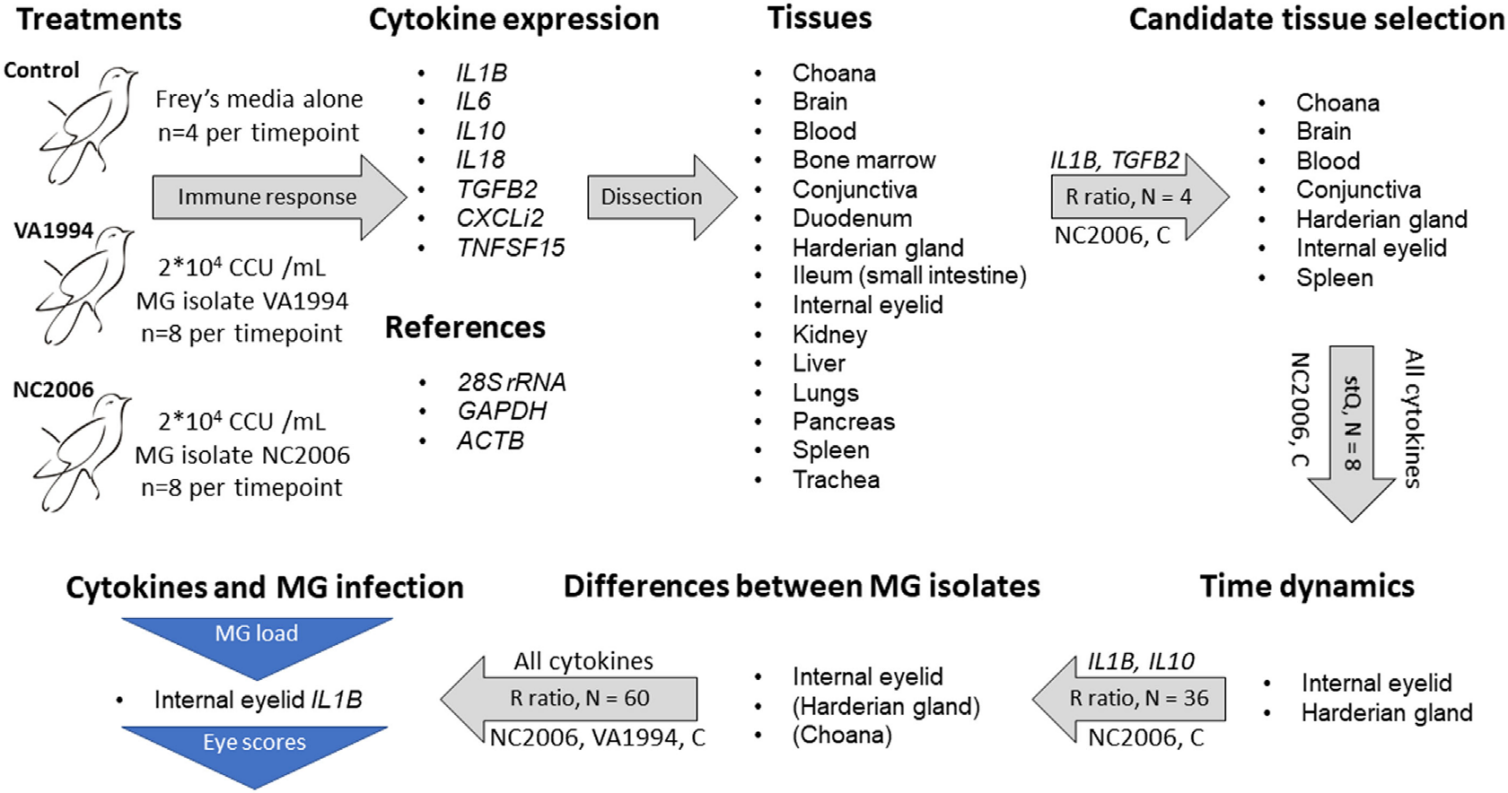
Design of the experiment and qPCR analysis. Successive steps are linked with gray arrows indicating procedures being done. The text above the arrows indicates the specific cytokine genes used for each step, and the text below the arrows indicates the treatment group(s) used for that step [control (C)]. The text inside the arrows indicates the sample size per tissue and the metric of gene expression used [relative expression (*R*) ratio (41) calculation and standardized expression quantity (stQ) (42, 43)]. The stQ quantification was used for analyses where statistical comparisons between treatments and controls were necessary, as this is not possible using the *R* ratio. Blue triangles indicate traits for which associations to cytokine expression were tested. For isolate differences in cytokine expression in Harderian gland and choana, only a subset of cytokines was examined, and thus those two tissues are in parentheses.

Finally, to compare the cytokine expression profiles of the response to VA1994 and NC2006 MG isolates in the data set of all experimental individuals, we again used the relative quantification based on relative expression ratio *R* (41), where, this time, ΔCqT was obtained as the difference in Cq values between control mean for the particular sampling time point and the treatment mean in the target gene (cytokine), and ΔCqR was the difference in Cq values between control mean for the particular sampling time point and the treatment mean in the reference gene (*28S rRNA*). Similarly, absolute quantification ratio was calculated as *A* = (CN_T_/CN_R_)_Treatment_/(CN_T_/CN_R_)_Control_, where CN_T_ is target gene copy number, CN_R_ is reference gene (*28S rRNA*) copy number; in controls we used mean copy number values for the particular sampling time point. Log base 2 values of *R* and *A* were used. A general overview of the gene expression data analysis procedure is shown in Figure 1.

### Statistics

All statistical analysis was performed using R software v. 3.4.0 (44) with a significance level of *P* = 0.05. Because cytokine expression data (both absolute and relative) often deviate from a normal distribution (tested using the Shapiro–Wilk normality test), we used Spearman’s rank correlation tests to reveal correlations between relative (stQ) and absolute (*A*) expression data. As the relative and aQ data were highly correlated (for all cytokine genes studied *P* = 0.001 and *r*_S_ > 0.84), we show only the results obtained for the relative expression data in the main text of the article and report analogous results obtained using absolute quantification in the Supplementary Material to provide quantification method-independent confirmation to our main results.

We used the non-parametric Wilcoxon rank sum test to test for differential gene expression (stQ or aQ) between control and NC2006-infected individuals screened across 7 tissues selected for further investigation after the initial cursory screening of 15 tissues in 2 individuals per treatment. To indicate consistency in the expression of the cytokine genes, principal component analysis (PCA) was done for the stQ and aQ data after log_2_-transformation. The first three principal component scores were added to a correlation matrix prepared using Pearson’s correlations with Holm’s adjusted *P*-values. A heatmap showing variation in cytokine gene expression was constructed based on log_2_-transformed stQ and aQ quantities in R software using the “heatmap” function of the “stats” library, with the Unweighted Pair Group Method with Arithmetic Mean selected as the clustering algorithm.

After this second and more in-depth round of screening of seven house finch tissues, we focused on a subset of two selected tissues (internal eyelid and HG) to examine the temporal dynamics of cytokine expression responses to MG and the differences between MG isolates. We used generalized linear models (GLMs) with cytokine expression ratio *R* or *A* as the response variable. For these GLMs, the non-Gaussian response variables were log_2_-transformed to achieve residual normality and tested against selected factors as explanatory variables: tissue or MG isolate (depending on analysis), time point, sex, and all two-way interactions. Because there was little variability in size and mass and the treatment groups and time point groups did not show any significant differences with respect to size or mass or mass change over the experiment (all *P* > 0.05), these variables were excluded from the full models tested in this study. We tested the significances of the explanatory variables both in full models and in minimum adequate models (MAMs; i.e., models with all terms either significant, *P* ≤ 0.05, or marginally non-significant, *P* < 0.10) that were obtained by backward eliminations of particular terms from the full model. Candidate models were compared based on the change in deviance with an accompanied change in degrees of freedom (ANOVA) using *F* statistics. Tukey’s *post hoc* tests were used to test for differences between individual time points or MG isolates.

A two-sample *t*-test was used to compare relative *IL1B* and *IL10* expression (log_2_*R*) induced by the two MG isolates in upper respiratory tract at DPI 6. Relative *IL1B* and *IL10* expression (log_2_*R*) comparisons between upper respiratory tract, internal eyelid and HG were done using linear mixed-effects models (LMMs), where log_2_*R* data (response variable) were tested against MG isolate, tissue type, sex and all two-way interactions (explanatory variables with fixed effects). Individual identity was included into the models as a random effect and we used backward elimination of fixed effects to obtain the minimum adequate models.

For testing the association between *IL1B* expression and conjunctival MG loads, *IL1B* log_2_*R* data were used as the response variable in a GLM containing endpoint MG quantities (log_10_MG), MG isolate, sampling time point (DPI), sex, and two-way interactions of the factors as explanatory variables. In an analogous GLM, we tested for an association between total eye scores and *IL1B* expression using the quasipoisson residual distribution. Here, the endpoint total eye scores were used as the response variable, and we included *IL1B* log_2_*R* data, MG isolate, sampling time point (DPI), sex and two-way interactions of the factors as explanatory variables in the model. Again, we used a backward elimination method (as described earlier) to obtain the minimum adequate models.

## Results

### Tissue-Specific Variation in Cytokine Expression Response to MG

To identify candidate tissues for further research, we first compared the relative expression ratios *R* based on two NC2006-infected and two control individuals for two selected genes (*IL1B* and *TGFB2*) across the whole panel of 15 tissues. Our results (Figure S1 in Supplementary Material) show highest *R* in at least one of these genes in blood, brain, conjunctiva, HG, choana and internal lid; we thus selected these six tissues for further analysis. Spleen was also included in further analysis as a standardly investigated lymphatic tissue for the purpose of comparison.

**Figure 2:**
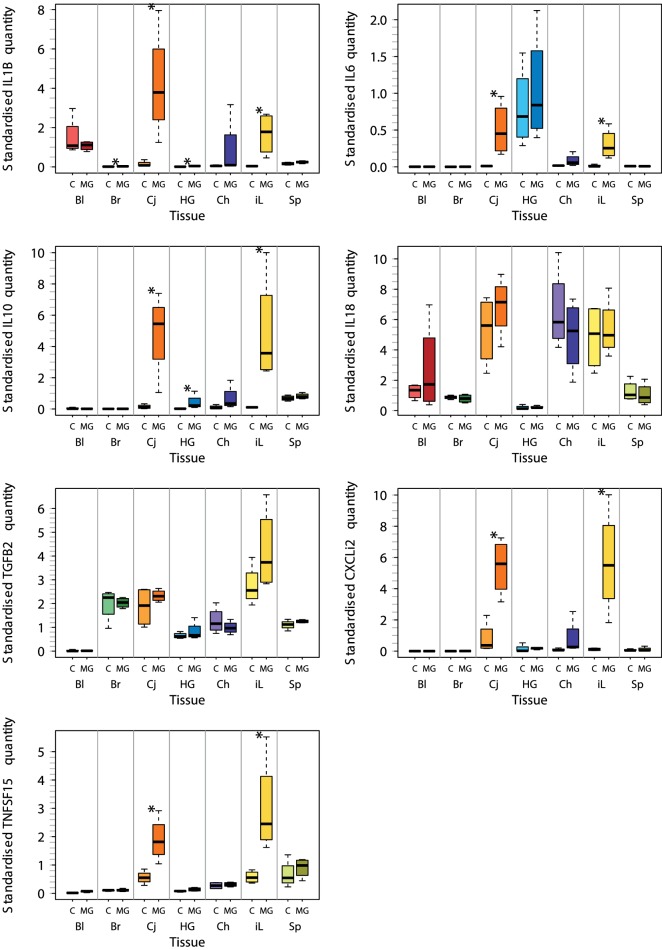
Tissue-specific differential cytokine gene expression in *Mycoplasma gallisepticum* (MG)-infected house finches versus uninfected control (C) on day 6 postinoculation (DPI 6). The boxplots show the median (line), the upper and lower quartiles (the box), and the range (dotted lines) of relative cytokine quantity standardized on *28S rRNA* expression (stQ). Tissue types are shown on the *x* axis highlighted with color: red—blood (Bl), green—brain (Br), orange—conjunctiva (Cj), blue—Harderian gland (HG), purple—choana and upper respiratory tract (Ch), yellow—nictitating membrane = internal eyelid (iL), light green—spleen (SP). Treatment type: C, control (light colors); MG, inoculation with MG isolate NC2006 (dark colors). Asterisks indicate significant difference in gene expression in the tissue (Wilcoxon test, *P* < 0.050).

**Figure 3:**
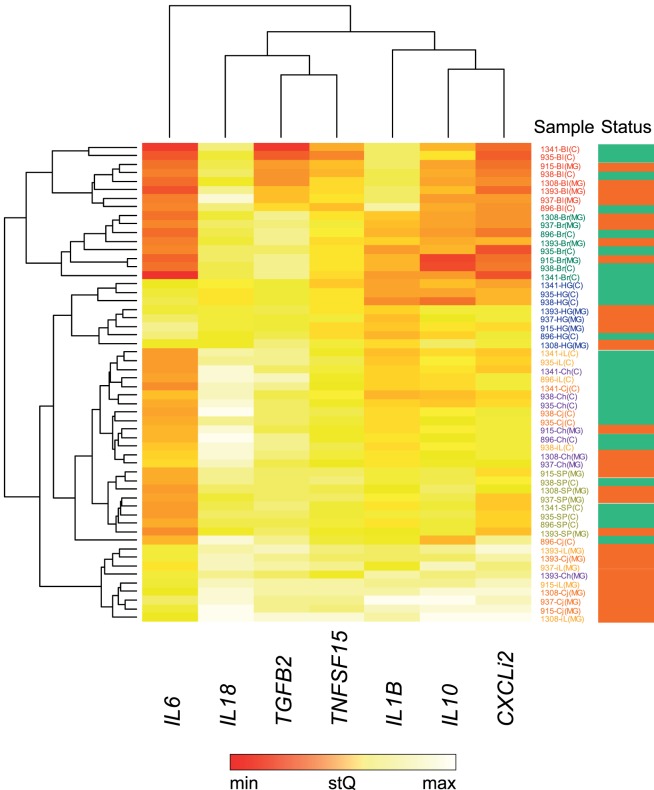
Heatmap of differential cytokine gene expression in *Mycoplasma gallisepticum* (MG)-infected (red bar, far right) and control (green bar, far right) house finches on day 6 postinoculation (DPI 6). For this analysis, all MG-infected birds received the NC2006 isolate. The heatmap is based on relative cytokine quantity standardized on *28S rRNA* expression (stQ). Gene names are shown at the bottom of the chart. Low gene expression is indicated in red; high gene expression in white. Tissue type is highlighted with color of the sample label: red—blood (Bl), green—brain (Br), orange—conjunctiva (Cj), purple—choana and upper respiratory tract (Ch), blue—Harderian gland (HG), light orange—nictitating membrane = internal eyelid (iL), light green—spleen (SP). Dendrograms showing the clustering of the cytokine expression patterns were constructed using Unweighted Pair Group Method with Arithmetic Mean method.

For these seven selected tissues, we analyzed the expression of all seven investigated cytokine genes in four control individuals and four NC2006-infected individuals at DPI 6 to identify the genes differentially expressed in individual tissues during MG infection. The cytokine standardized relative quantity data (stQ) show significant differential expression of *IL1B* in brain, conjunctiva, HG and internal eyelid, of *IL6* in conjunctiva and internal eyelid, of *IL10* in conjunctiva, HG and internal eyelid, and of *CXCLi2* and *TNFSF15* in conjunctiva and internal eyelid (Figure 2; see Figure S2 and Tables S5 and S6 in Supplementary Material for analogous results obtained based on aQ data, *A*). No differential expression was indicated for spleen and only marginally non-significant changes in inflammatory cytokine expression were revealed in blood and upper respiratory tract. No differential expression was revealed for *IL18* or *TGFB2* in any tissue. While there was low consistency in the cytokine expression patterns across distinct tissues (except for conjunctiva and internal eyelid; Figure 3; Figure S3 in Supplementary Material), the expression of different cytokine genes was highly correlated within individual tissue samples (Table S7 in Supplementary Material; PCA: cumulative proportion of variance explained by PC1–PC3 = 0.898). Based on these results and prior work in this system (28), we selected pro-inflammatory *IL1B* and anti-inflammatory *IL10* as the candidate genes, and internal eyelid and HG as the target tissues, for investigation of the temporal dynamics of cytokine expression.

### Temporal Dynamics of the Cytokine Expression Response to MG

**Figure 4:**
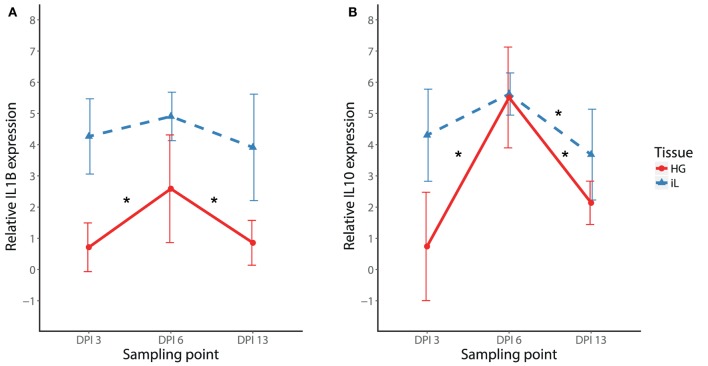
Temporal dynamics of *IL1B* **(A)** and *IL10* **(B)** expression in response to MG-NC2006 infection in selected tissues across three different time points. The data are shown as relative expression ratio (*R*; i.e., *28S rRNA*- and control-normalized relative expression quantities) mean ± SD. Red circles represent values in Harderian gland, and blue triangles indicate values in internal eyelid. Abbreviation: DPI, day postinoculation. Asterisks mark significant differences between DPI in the respective tissues (Tukey’s *post hoc* test *P*_adj_ < 0.050).

To initially compare temporal patterns of cytokine expression in internal eyelid and HG in MG infected birds, we compared *IL1B* and *IL10* expression changes in NC2006 isolate-inoculated birds. Our results show significant effects of both tissue type and DPI for both genes (relative quantification: Figure 4; Table S8 in Supplementary Material; for absolute quantification, see Supplementary Material). Although in both genes there is apparent peak of the response at DPI 6 in both tissues, Tukey’s *post hoc* tests showed significant differences between the sampling time points in internal eyelid only in *IL10* (DPI 6–DPI 13: *P* = 0.023), while in HG, DPI 6 was significantly different from the other sampling time points for both genes (*IL1B*: DPI 6–DPI 3: *P* = 0.018, DPI 6–DPI 13: *P* = 0.030; *IL10*: DPI 6–DPI 3: *P* = 0.001, and DPI 6–DPI 13: *P* < 0.001).

### Differences between Cytokine Expression Responses to Original and Evolved MG Isolate

**Figure 5:**
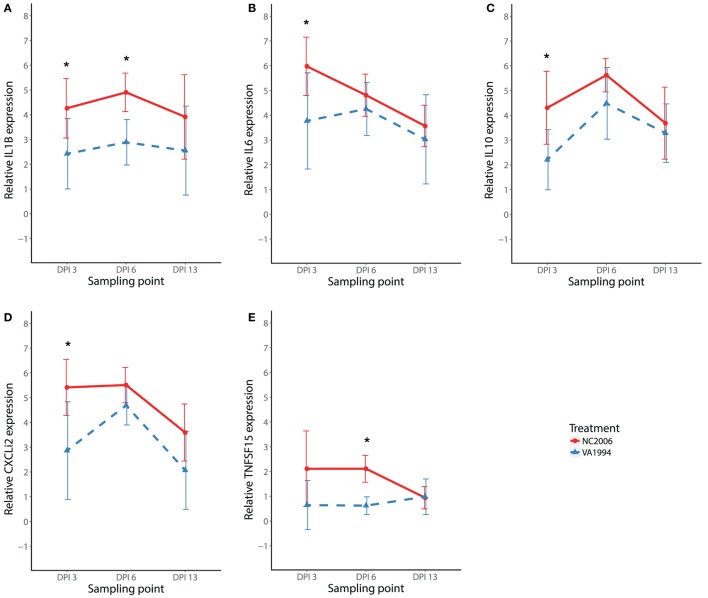
Effects of experimental infection with *Mycoplasma gallisepticum* isolates VA1994 and NC2006 on *IL1B* **(A)**, *IL6* **(B)**, *IL10* **(C)**, *CXCLi2* **(D)**, and *TNFSF15* **(E)** expression in internal eyelid (nictitating membrane) across three different time points. The data are shown as *28S rRNA*- and control-normalized mean ± SD relative expression quantities. Blue triangles indicate VA1994 isolate data, and red circles represent NC2006 isolate data. Asterisks indicate significant difference in gene expression between isolates at that time point (Tukey’s *post hoc* test, *P* < 0.050). Sampling points: DPI 3, 3 days post inoculation; DPI 6, 6 days post inoculation; DPI 13, 13 days post inoculation.

To compare the immune responses triggered by distinct MG isolates (VA1994 and NC2006), we selected the internal eyelid as a primary model tissue, because here the strength of cytokine expression relative to controls was most prominent. In *IL1B, IL10, IL6, CXCLi2*, and *TNFSF15*, we found significant effects of MG isolate on cytokine expression quantified using both relative (Table 2; Figure 5) and absolute approaches (Supplementary Material). Generally, the evolved NC2006 isolate triggered stronger cytokine responses than the original VA1994 isolate, though the *post hoc* significance of isolate differences varied across genes and time points, with the strongest differences between isolates generally occurring earlier in infection (Figure 4). In most cases, we also detected significant effects of sampling time point. However, there was no significant effect of MG isolate or sampling time point on expression of *IL18* and *TGFB2* in internal eyelid in house finches (relative and absolute quantification data; all terms *P* > 0.05).

**Table 2:** Minimum adequate models (MAMs) for effects of infection with *Mycoplasma gallisepticum* isolates VA1994 and NC2006 on house finch expression of cytokines *IL1B, IL6, IL10, CXCLi2*, and *TNFSF15* in internal eyelid (nictitating membrane) across three different time points.

MAM/variable	Df	*F*	*P*
Log_2_*R*(IL1B) ~ treatment	1/46	17.88	<0.001
Log_2_*R*(IL6) ~ treatment + DPI	3/44	5.63	0.002
Treatment	1/44	6.70	0.013
DPI	2/44	5.09	0.010
Log_2_*R*(IL10) ~ treatment + DPI	3/44	8.50	<0.001
Treatment	1/44	9.33	0.004
DPI	2/44	8.09	0.001
Log_2_*R*(CXCLi2) ~ treatment + DPI	3/44	12.41	=0.001
Treatment	1/44	16.21	<0.001
DPI	2/44	10.51	<0.001
Log_2_*R*(TNFSF15) ~ treatment + DPI	5/42	4.55	0.002
Treatment	3/42	6.87	0.001
DPI	4/42	2.35	0.070
Treatment: DPI	2/42	3.63	0.035

*Based on the relative expression ratio (*R*) data (i.e., *28S rRNA*- and control-normalized relative expression quantities)*.

*Treatment, inoculation with VA1994 or NC2006; DPI, days postinoculation; IL, interleukin; TNFSF, tumor necrosis factor superfamily members*.

**Figure 6:**
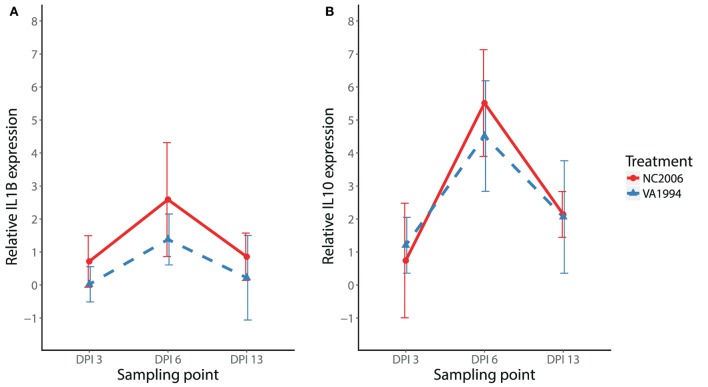
Effects of experimental infection with *Mycoplasma gallisepticum* isolates VA1994 and NC2006 on *IL1B* **(A)** and *IL10* **(B)** expression in Harderian gland across three different time points. The data are shown as *28S rRNA*- and control-normalized mean ± SD relative expression quantities. Blue triangles indicate birds inoculated with VA1994 isolate, and red circles indicate birds inoculated with NC2006. Sampling points: DPI 3, 3 days post inoculation; DPI 6, 6 days post inoculation; DPI 13, 13 days post inoculation.

We also compared isolate-specific patterns of expression (original VA1994 versus evolved NC2006) for a subset of cytokines (*IL1B* and *IL10*) in HG. Similar to the results from internal eyelid, the NC2006 isolate triggered stronger *IL1B* responses than the original VA1994 isolate in HG (MG isolate *F*_1/44_ = 7.03, *P* = 0.011; stp *F*_2/44_ = 10.33, *P* < 0.001; MAM: *F*_3/44_ = 9.23, *P* = 0.001; Figure 6A). However, for the anti-inflammatory cytokine *IL10*, expression in HG was dependent only on sampling time point (MAM: *F*_2/45_ = 29.78, *P* = 0.001; Figure 6B) and not the MG isolate.

**Figure 7:**
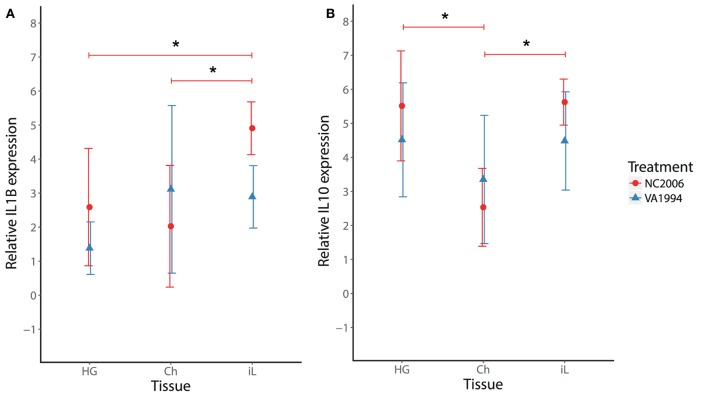
Effects of experimental infection with *Mycoplasma gallisepticum* isolates VA1994 and NC2006 on *IL1B* **(A)** and *IL10* **(B)** expression in selected tissues at the time of peak cytokine expression [day 6 post inoculation (DPI 6)]. The data are shown as *28S rRNA*- and control-normalized mean ± SD relative expression quantities. Blue triangles indicate VA1994 isolate data, and red circles represent NC2006 isolate data. Asterisks above connecting lines indicate significant difference in gene expression between tissues (Tukey’s *post hoc* test, *P* < 0.050). Tissues: HG, Harderian gland; Ch, upper respiratory tract and choana; iL, internal eyelid (nictitating membrane).

Because MG infection in chickens induces cytokine responses in the upper respiratory tract (27), we also checked for isolate-specific differences in *IL1B* and *IL10* stimulation in the upper respiratory tract at the time of the peak cytokine expression response (DPI 6) and compared the cytokine expression in this tissue with cytokine expression in HG and internal eyelid. Although we found significant effects of both tissue and MG isolate on *IL1B* and *IL10* expression across the three tissues (LMM analysis; Table S12 in Supplementary Material), we did not detect any significant differences between the MG isolates in either *IL1B* or *IL10* in the upper respiratory tract in particular (two-sample *t*-test, in both cases *P* > 0.05; Figure 7). Thus, isolates VA1994 and NC2006 activate similar pro-inflammatory cytokine expression responses in the upper respiratory tract, but as shown earlier, NC2006 triggers significantly stronger immune activation in internal eyelid and HG (for *IL1B* alone) than VA1994 (Figure 7A). When analyzed as tissue-specific differences within individuals, there is no difference in *IL1B* and *IL10* expression between upper respiratory tract, HG and internal eyelid for VA1994 (Tukey’s test, in all cases *F*_adj_ > 0.05); however, in the case of NC2006, cytokine expression responses are significantly stronger in internal eyelid/HG compared with the upper respiratory tract (Tukey’s *post hoc* test, *IL1B*: Ch-HG *P*_adj_ > 0.05, iL-HG *P*_adj_ = 0.023, iL-Ch *P*_adj_ = 0.005, *IL10*: Ch-HG *P*_adj_ < 0.001, iL-HG *P*_adj_ > 0.05, iL-Ch *P*_adj_ < 0.001).

### Relationship between Cytokine Expression and Intensity of MG Infection

**Figure 8:**
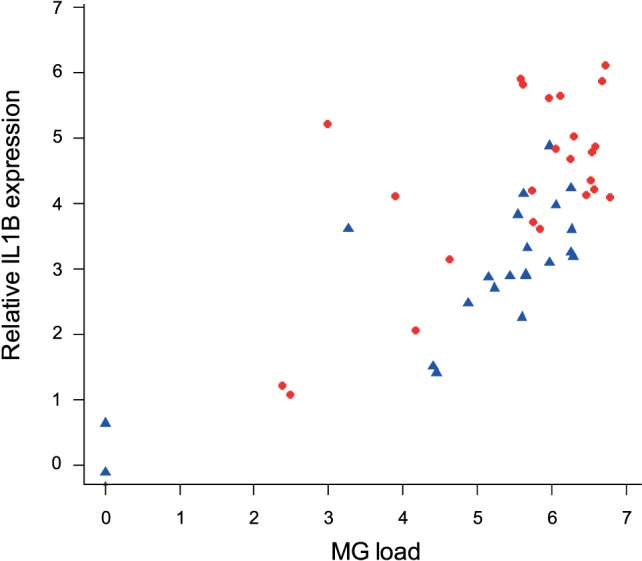
Association between conjunctival MG loads at the time of examination (which varied for each batch collected at one of three sampling time points) and *IL1B* expression in internal eyelid. The data on *IL1B* expression are shown as *28S rRNA*- and control-normalized relative expression quantities (log_2_*R*). Because isolate was significant in the statistical model, points are labeled by MG isolate (blue circles = VA1994 and red triangles = NC2006).

To examine the association between MG infection intensity (MG loads in the conjunctiva at the time of sampling, which differed by batch) and cytokine expression in periorbital tissues, we focused on *IL1B* as the cytokine that showed the most consistent differences between isolates. We found strong effects of conjunctival MG load on internal eyelid *IL1B* relative expression (*F*_1/45_ = 69.37, *P* = 0.001), with higher *IL1B* relative expression in birds with higher conjunctival MG burdens (Figure 7). We also found notable effect of MG isolate (*F*_1/45_ = 18.53, *P* = 0.001), with higher average pathogen loads in birds inoculated with NC2006 (MAM: *F*_2/45_ = 56.90, *P* = 0.001; Figure 8).

**Figure 9:**
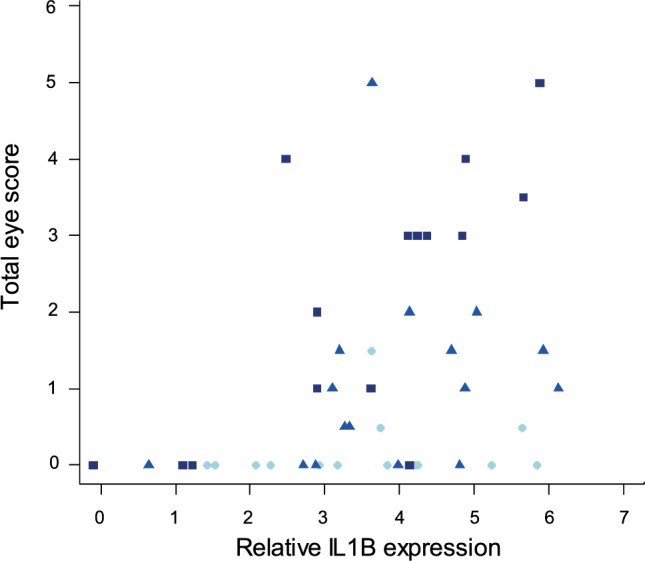
Relationship between *IL1B* expression in internal eyelid and total eye scores (index of MG pathogenicity) at the time of sampling (which varied for each batch collected at one of three sampling time points). The data on *IL1B* expression (explanatory variable) are shown as *28S rRNA*- and control-normalized relative expression quantities (log_2_*R*). Because sampling time point was significant in the minimum adequate model, points are labeled by sample date (light blue = DPI 3, blue = DPI 6, and dark blue = DPI 13).

Finally, to examine the relationship between pro-inflammatory cytokine expression in internal eyelid and MG pathogenicity, we analyzed the interaction between *IL1B* and total eye scores (representing the visible pathological effects of MG infection) at the time of sampling. Our results show significant associations between *IL1B* expression (*F*_1/44_ = 13.32, *P* < 0.001), sampling time point (*F*_2/44_ = 12.37, *P* = 0.001), and total eye scores (MAM: *F*_3/44_ = 12.66, *P* = 0.001; Figure 9), such that birds with higher *IL1B* expression had significantly higher total eye scores.

## Discussion

*Mycoplasma gallisepticum*-infected house finches show several systemic changes in immunological traits, including alterations in blood leukocyte profiles (22) and antibody production (24). In this study, we characterized the tissue distribution and temporal dynamics of cytokine responses to MG infection, showing that cytokine expression changes are strongest in periocular tissues, and peak between days 3 and 6 postinoculation (PI). Furthermore, we showed that cytokine responses are significantly stronger following inoculation with an evolutionarily derived lineage of MG, and that the load of MG infection among individuals directly predicts the degree of *IL1B* expression, which is associated with the severity of mycoplasmal conjunctivitis (an index of MG pathogenicity).

The capacity of MG to trigger expression of pro-inflammatory cytokines has been previously shown in both poultry (27, 45, 46) and human (47) cells and tissues. Here, we first used a subset of individuals to characterize the tissue distribution of cytokine expression on day 6 postinoculation in house finches. For this analysis, we chose to only examine the most virulent isolate of MG to maximize our likelihood of detecting differential expression. We detected significant upregulation of five (*IL1B, IL10, IL6, CXCLi2*, and *TNFSF15*) of the seven examined cytokines in conjunctiva and nictitating membrane (internal eyelid), and significant upregulation of *IL1B* and *IL10* in HG. In birds, the conjunctiva and the HG are parts of the eye-associated secondary lymphoid tissue (48). Being colonized with large number of T cells and B cells, the HG plays an important role in the regulation of ocular immunity, including antibody and inflammatory responses to conjunctival pathogens. The nictitating membrane, on the other hand, is primarily a non-lymphoid tissue, that is, however, rapidly infiltrated with leukocytes upon MG infection.

While upregulation of key cytokines in the periocular tissues during MG infection is not surprising, we also observed significant upregulation of *IL1B* expression in the brain. IL1B experimentally administrated to the brain as well as released in the periphery during infection can elicit vertebrate sickness behaviors and impair memory (49, 50). Our results support the view that, like in mammals (51), IL1B may act in the avian brain as an important mediator of the acute phase response, which in house finches leads to fever and severe lethargy associated with MG infection (12, 28). The mechanism behind this relationship, however, awaits verification in the house finch–MG system.

There was only low consistency in the cytokine expression patterns across distinct tissues, and we found no evidence for significant differential cytokine expression in any other tissues aside from the brain and periocular tissues, including spleen, upper respiratory tract, or blood. Although this result could be affected by the limited sample size used for the investigation of the tissue distribution of cytokine expression (*n* = 4 per treatment), overall, our results clearly indicate that differential cytokine expression responses during MG infection are strongest in periorbital tissues (conjunctiva, internal eyelid, and HG). Finally, although the expression of most inflammatory cytokine genes was highly correlated within individual tissues, we did not detect differential expression of *IL18* or *TGFB2* in any tissue examined, suggesting that these cytokines do not respond to MG infection. This pattern is consistent with house finch cytokine regulation toward a type 1 adaptive immune response that is mediated by non-specific inflammation leading to induction of Th1 cell activation.

After determining which genes and tissues would be most relevant for further study, we examined the temporal dynamics of cytokine expression in internal eyelid and HG (two distinct periorbital immune tissues), focusing on pro-inflammatory *IL1B* and anti-inflammatory *IL10*. For both of these cytokines, responses peaked at day 6 postinoculation, but this peak was only significant in both genes for HG, where overall expression was generally lower than in internal eyelid. In poultry, *IL1B* expression responses to MG inoculation appear to be strongest between day 4 and day 8 PI (27). This is consistent with our further findings on the temporal dynamics in most other pro-inflammatory cytokines (*IL6, CXCLi2*, and *TNFSF15*), where the expression in internal eyelid exhibits similar patterns of early upregulation (DPI 3–6) followed by a decline in cytokine expression at our latest sampling point examined (DPI 13). Nevertheless, our multiple-isolate analysis showed that the temporal dynamics of responses varied to some extent with the strain of MG used. Future work with higher temporal sampling resolution and larger sample sizes will help to shed further light on strain-specific temporal dynamics of cytokine expression in house finches.

A primary goal of our study was to examine how pathogen evolution affects cytokine expression responses in its host. Various MG strains, including those from the house finch–MG clade, differ in their surface antigens, which may cause variation in their interaction with the immune system of the host (16, 52). Because MG has evolved to become significantly more virulent since its initial emergence in house finches (18), we characterized cytokine responses to an original field isolate (VA1994) collected the year that MG was first detected in house finches, and a more evolved isolate (NC2006) shown to be significantly more virulent in house finches. These two isolates are closely related (11) but show notable genomic differences (19) and produce markedly distinct host responses and epidemiological parameters, with NC2006 producing significantly higher conjunctival pathogen loads and disease severity (18), stronger IgG and IgA responses (9), and faster rates of transmission than that of VA1994 (53). For all five of the cytokine genes differentially expressed on day 6 post-MG inoculation in periocular tissues (*IL1B, IL6, IL10, CXCLi2*, and *TNFSF15*), we found significantly stronger cytokine responses in birds inoculated with NC2006 relative to those inoculated with VA1994 in internal eyelid (and for *IL1B* in HG). These results are consistent with the stronger stimulation of humoral responses by the NC2006 isolate relative to the VA1994 isolate detected in prior work (9). We did not detect any significant changes in cytokine expression in response to MG infection in the upper respiratory tract, and we did not detect any differences between the MG isolates in either *IL1B* or *IL10* expression in this tissue. Hence, our results suggest that the house finch–MG strains have evolved in their capacity to specifically elicit pro-inflammatory cytokine expression in periorbital tissues and not in other tissues, such as the upper respiratory tract. MG was previously shown to adapt to its passerine host, resulting in milder virulence for more evolved house finch–MG isolates in the original poultry host (54, 55), but increased virulence in the novel house finch host, where the disease has become established (18, 56). Although our results are strongly suggestive of an evolutionary change in cytokine expression linked with increased virulence, multiple evolved (and virulent) isolates are needed to definitively link strain-level changes in virulence with host cytokine expression responses.

We also leveraged individual variation in the degree of pathogenicity (eye lesion score) and infection intensity (conjunctival pathogen load) at the time of euthanasia to further link host immune responsiveness to virulence, as has been done in prior studies with humoral responses (9). We show that birds with higher expression of *IL1B* also had significantly higher eye lesion scores and conjunctival pathogen loads. Together, these results suggest that prolonged house finch pro-inflammatory cytokine responses are likely not protective during MG infection, but instead may underlie the degree of pathology experienced by hosts. Thus, although evolution of a protective immune response to MG has been reported in house finches (4, 26, 57), mycoplasmal conjunctivitis *per se* appears to be largely immunopathological in house finches, with important implication for host–pathogen coevolution (58). Experimental manipulations of pro-inflammatory cytokine signaling in the house finch–MG system are, nonetheless, needed to confirm the causality underlying the detected associations.

There are several documented examples of animal diseases where overactivation of immune cytokine signaling is responsible for immunopathology (59–61). In poultry, overly strong inflammation is likely a cause of some of the pathologies associated with mycoplasmosis (62). Although much of the recent knowledge on cytokine regulation of inflammation comes from mammalian studies (63), present evidence from birds, mainly from the domestic chicken (31), suggests that (although equipped with slightly different sets of cytokines) the basic functions of the most essential cytokines may be conserved within amniotes. In house finches, this has been confirmed for IL1B, where its conserved function was demonstrated in splenocytes (38). Our results, combined with prior research (28), suggest that the degree of inflammation is a key trait underlying house finch responses to this disease. Thus, any factors that suppress inflammation, such as anti-inflammatory cytokines, Treg cells or circulating immunosuppressing stress hormones levels, may be key in limiting the severity of disease, and thus, the fitness effects on house finches. In fact, Love et al. (64) showed that preinfection glucocorticoid (in this case, corticosterone) concentrations in male house finches were associated with reduced inflammation and pathogen load, suggesting that dampened inflammation may be a key mechanism of resistance or tolerance in this system.

Pathogens have been shown to use many different means to manipulate host immunity for the purpose of increasing their transmission rate (65, 66). This manipulation may include downregulation, as well as upregulation of host inflammatory immunity that may be used by the pathogen to increase permeability of host tissues and facilitate transmission. While Ganapathy and Bradbury (67) previously reported temporary T-cell suppression at 2 weeks post MG infection in chickens, it is possible that in house finches, MG manipulates its host toward more intense and/or prolonged pro-inflammatory gene expression in the periocular tissues. In house finches experimentally inoculated with MG, enhanced pathology (i.e., higher eye scores) leads to a higher proportion of conjunctival MG deposited onto bird feeders (68), likely due to exudate or swelling enhancing pathogen deposition into the environment. Thus, prolonged or enhanced expression of pro-inflammatory cytokines may have important fitness benefits for MG by enhancing host pathologies that contribute to transmission. However, experiments directly manipulating cytokine levels are needed to causally test this hypothesis.

Altogether, our results show that increased virulence of an evolutionarily derived MG isolate is associated with increased periocular expression of pro-inflammatory cytokines. Although our experiment cannot confirm the direction of causality underling this association, immunopathology induced by this inflammation might explain the mechanism of maladaptation of house finch immunity to MG. Given the demonstrated fitness costs of conjunctivitis for free-living house finches (69), future research should examine whether house finch populations with distinct coevolutionary histories with MG differ in their inflammatory cytokine responses to this pathogen, which would suggest that host evolution is also influencing house finch cytokine responses. Overall, future studies that simultaneously examine evolutionary variation in both host and pathogen will be critical to dissecting the distinct contributions of each coevolutionary player to house finch pro-inflammatory cytokine responses during MG infection.

## Ethics Statement

This study was carried out in accordance with the recommendations of federal (USFWS permit MB158404-1) and state (VDGIF permit 050352) agencies. The protocol was approved by the Virginia Tech’s Institutional Animal Care and Use Committees.

## Author Contributions

MV and DH designed the research; MV, AL, LK, and DH performed the research procedures; RD provided RNA-seq data; MV and DH analyzed the data; and MV, RD, and DH prepared the manuscript. All the authors contributed with their comments to the conception of the work and final version of the manuscript.

## Conflict of Interest Statement

The authors declare that the research was conducted in the absence of any commercial or financial relationships that could be construed as a potential conflict of interest.
