# Supplementary material for: Differing House Finch Cytokine Expression Responses to Original and Evolved Isolates of Mycoplasma gallisepticum
Source: Front Immunol. 2018 Jan 22;9:13. doi: 10.3389/fimmu.2018.00013 (PMC5786573; doi:10.3389/fimmu.2018.00013)
Supplement: Supplementary file 1 [file Presentation_1.PDF]

Supplementary Material

Title: Differing house finch cytokine expression responses to original and evolved isolates of a newly emerged bacterial pathogen

Authors: **Michal Vinkler*, Ariel E. Leon, Laila Kirkpatrick, Rami A. Dalloul and Dana M. Hawley**

*** Correspondence:** Michal Vinkler: michal.vinkler@natur.cuni.cz

1 Methods

1.1. Cankarex and sulfadimethoxine treatment

Upon capture, birds were first treated with Cankarex (0.25 g/L) for 5 days in drinking water to prevent overgrowth of *Trichomonas* parasites. Birds were then treated with sulfadimethoxine (starting at a high dose 0.496 mg/ml for 5 days, and dropping to low dose 0.26 mg/ml) to control intestinal coccidia infections for a minimum of 14 days post-capture.

1.2. Cytokine gene sequencing

Total RNA was extracted from spleen samples of 7 house finch individuals stored in RNAlater (Ambion, Cat. No. AM 7021) and frozen at -80°C using the High Pure RNA Tissue Kit v. 09 (Roche, Cat. No. 12033674001) according to manufacturer's instructions. After measuring concentration on NanoDrop 2000, equalised amounts of RNA were reverse-transcribed using the Transcriptor High Fidelity cDNA Synthesis Kit v. 09 (Roche, Cat. No. 05081955001) and the cDNA was stored at -20°C. PCR was then performed using the Multiplex PCR Kit (Qiagen, Cat. No. 206143) with primers (designed using the OligoAnalyzer web tool, Integrated DNA Technologies, Inc., Coralville, IA USA, based on partial sequences obtained through RNA-seq and canary reference) and cycling conditions described in Table S1. The electrophoresis-checked PCR products were cleaned-up using the ExoSAP kit (Affymetrix, Cat. No. 78250) and send for Sanger sequencing with the same primers to the Genomics Research Laboratory, Biocomplexity Institute, Virginia Tech.

Table S1. **Sex and age composition of treatment categories.** C = control, VA1994 = MG isolate VA1994, NC2006 = MG isolate NC2006, DPI = day post-inoculation, M = male, F = female, H = hatch-year individuals, U = unknown, N = the number of individuals in the study from each treatment, timepoint, sex, and age combination. Note that age also reflects capture date, as all birds known to be hatch-year were captured in June-July 2015 and all those of unknown age were captured in December 2015.

Treatment	DPI	Sex	Age	N
C	3	M	H	2
C	3	M	U	0
C	3	F	H	1
C	3	F	U	1
C	6	M	H	1
C	6	M	U	1
C	6	F	H	1
C	6	F	U	1
C	13	M	H	1
C	13	M	U	2
C	13	F	H	1
C	13	F	U	0
VA1994	3	M	H	2
VA1994	3	M	U	2
VA1994	3	F	H	1
VA1994	3	F	U	3
VA1994	6	M	H	1
VA1994	6	M	U	3
VA1994	6	F	H	2
VA1994	6	F	U	2
VA1994	13	M	H	3
VA1994	13	M	U	3
VA1994	13	F	H	2
VA1994	13	F	U	0
NC2006	3	M	H	2
NC2006	3	M	U	2
NC2006	3	F	H	1
NC2006	3	F	U	3
NC2006	6	M	H	2
NC2006	6	M	U	2
NC2006	6	F	H	3
NC2006	6	F	U	1
NC2006	13	M	H	4
NC2006	13	M	U	1
NC2006	13	F	H	1
NC2006	13	F	U	2

Table S2. **Polymerase chain reaction (PCR) conditions and primers used for sequencing the regions of interest in the cytokine genes.** Ta – annealing temperature, Tm – melting temperature. Conditions used for the PCR amplification: 1) 95°C 15min., 2) (94°C 30sec., Ta 90sec., 72°C 90sec.)×35, 3) 72°C 10min.

Gene	Primer name	Primer sequence	Tm	Ta	Product length
ACTB	HaMeACTB-1-F	GTGCTGTCTTCCCATCCATC	55.7°C	55	1583 bp
	HaMeACTB-1-R	CCACATACTGGCACCACTTC	55.8°C		
GAPDH	aviGAPDH_F1	TCTGGCAAAGTCCAAGTG	52.5°C	60	1125 bp
	aviGAPDH_R1	CAGATCAGTTTCTATCAGCCTC	52.6°C		
IL1B	HaMeIL1B-1-F	GCTGACAGTGACCTTGGCAG	58.6°C	62	545 bp
	HaMeIL1B-1-R	GCAATGTTCACCTGGTCTGG	56.4°C		
IL6	HaMeIL6-1-F	AACAACCTCAACCTCCCCAAG	57.3°C	60	402 bp
	HaMeIL6-1-R	TCAAACACTGAAACTCCTGGTG	55.2°C		
IL8L (*CXCLi2*)	HaMeCXCLi2-1-F	AAACTCGTAGCTGTCCTGGC	57.2°C	60	224 bp
	HaMeCXCLi2-1-R	TCCAAGCACACCTCTTTGCC	58.1°C		
IL10	HaMeIL10-1-F	GCCCGCCAAGCTCAAAGAGCT	63.0°C	65	383 bp
	HaMeIL10-1-R	AGTTGTCAAACTCTCCCATGGCCT	60.5°C		
IL18	HaMeIL18-1-F	ACAGACCAGGAGATGCAATC	54.6°C	60	246 bp
	HaMeIL18-1-R	GGAGCAGCAAGATGTAAATGTC	54.1°C		
TGFB2	HaMeTGFB2-1-F	GCACTGCTATCTCCTGAGCG	57.8°C	60	1671 bp
	HaMeTGFB2-1-R	GGGACAGATACAGCAACTCCAC	57.4°C		
TNFSF15	HaMeTNFSF15-2-F	TGCTGCTGCTCATGCTG	56.2°C	62	458 bp
	HaMeTNFSF15-1-R	GTTCTCACTGAGGGTCTTGG	55.0°C		

1.3. qPCR assay

Conditions used universally for the Reverse transcription quantitative polymerase chain reaction (RT-qPCR) in all assays: 1) 50°C 10min., 2) 95°C 3min., 3) (95°C 15sec., 60°C 60sec.)x40. iTaq™ Universal Probes One-Step Kit (BioRad, Cat. No. 172-5140), Final primer concentration was 0.6 µM, final probe concentration was 0.125 µM. RNA template was diluted 1:5 for cytokine qPCRs, and 1:500 for *28SrRNA* qPCR.

Table S3. **Reverse transcription quantitative polymerase chain reaction (RT-qPCR) primers and probes.** E – PCR efficiency.

Gene	Primer name	Primer sequence	Prod. length	Probe name	Probe sequence	Probe orientation	E
28SrRNA	avi28SrRNA-F1	GGCGAAGCCAGAGGAAACT	62 bp	avi28SrRNA-P1	AGGACCGCTACGGACCTCCACCA	R	1.92
	avi28SrRNA-R1	GACGACCGATTTGCACGTC					
ACTB	HaMeACTB-F2	CATTGCTGACAGGATGCAG	102 bp	HaMeACTB-P1	CAAGATCATTGCCCCACCTGAGC	F	1.99
	HaMeACTB-R3	CCGATCCAGACAGAGTATTTG					
GAPDH	HaMeGAPDH-F1	CATCCTGGCATACACAGAG	100 bp	HaMeGAPDH-P1	CCATTCCTCCACCTTTGATGCGG	F	1.99
	HaMeGAPDH-R1	GTCGTTCAGTGCAATGCC					
IL1B	HaMeIL1B-F2	TGCTGGACAGAAAGTGAAGCT	110 bp	HaMeIL1B-P1	CAACATTGCTCTGTACCGTCCCCG	F	1.94
	HaMeIL1B-R3	GCTGGTAGCCCTTGATGC					
IL6	HaMeIL6-F2	CAGCGAAAACCAAAATGTTG	129 bp	HaMeIL6-P1	CAGAGCACCTGGCACGTACCATAAG	F	1.97
	HaMeIL6-R2	GTGTGGAGTGATTCCTGG					
IL8L	HaMeCXCLi2-F2	CAGTGCATAGCCACTCATTC	129 bp	HaMeCXCLi2-P1	CCGCTCTGTGTCAGCTTCACATCCT	R	1.99
(CXCLi2)	HaMeCXCLi2-R2	GCACACCTCTTTGCCATTC					
IL10	HaMeIL10-F1	AACCTCCTGCTGAACCTG	86 bp	HaMeIL10-P1	ATGAGACTCTGTCACAAATTCTTCACCTGT	F	1.89
	HaMeIL10-R2	ATGTGCTCCATGCTCCTG					
IL18	HaMeIL18-F2	TCAGTGTCCAGGTGGAAAAC	84 bp	HaMeIL18-P1	TCTCCACATTCCCTCTCACAGCACA	R	1.97
	HaMeIL18-R2	CTCCTTCCTTGAACCTCACG					
TGFB2	HaMeTGFB2-F3	GGCTCCATCACAGAGACAGG	119 bp	HaMeTGFB2-P1	CATTGTCCATGCTGTACCTTCGTACCTT	F	2
	HaMeTGFB2-R3	TCTTGCTTCAAGCTCCTCAC					
TNFSF15	HaMeTNFSF15-F3	GGGGCTCTCACTTTCTG	103 bp	HaMeTNFSF15-P1	CAGACACACTTTCCAGTGCAGAGAAGCC	F	1.95
	HaMeTNFSF15-R2	AGGTCCTGCCTCTTCAC					

Table S4. **Synthetic DNA standards (gBlocks) used for the RT-qPCR assays.**

Gene	Standard name	Length	Standard sequence (5'-3')
28SrRNA	avi28SrRNA-S1	126	CCCGAAAGATGGTGAACTATGCCTGGGCAGGGCGAAGCCAG<br>AGGAAACTCTGGTGGAGGTCCGTAGCGGTCCTGACGTGCAA<br>ATCGGTCGTCCGACCCGGGTATAGGGGCGAAAGACTAATCG<br>AAC
ACTB	HaMeACTB-S1	150	GGTGGTACCACAATGTACCCTGGCATTGCTGACAGGATGCA<br>GAAGGAGATCACAGCCCTGGCACCCAGCACAATGAAAATCA<br>AGATCATTGCCCCACCTGAGCGCAAATACTCTGTCTGGATC<br>GGGGGCTCCATCCTGGCCTCCCTGTCC
GAPDH	HaMeGAPDH-S1	150	GGGTAGTGAAGGCTGCTGCTGATGGGCCCCTGAAGGGCATC<br>CTGGCATACACAGAGGACCAGGTTGTCTCCTGTGACTTCAA<br>TGGTGATAGCCATTCCTCCACCTTTGATGCGGGTGCTGGCA<br>TTGCACTGAACGACCATTTTGTCAAGC
IL1B	HaMeIL1B-S1	150	CTGCACCTGCAGGGACCCTCTGCTGGACAGAAAGTGAAGCT<br>CAACATTGCTCTGTACCGTCCCCGGTCATCGCAGGGCGCTC<br>CAGGGTCTGGGAGGGTGCCAGTGGCATTGGGCATCAAGGGC<br>TACCAGCTCTACATGTCATGTGTGATG
IL6	HaMeIL6-S1	150	GAAACTTTTATCAGCGAAAACCAAAATGTTGAATCACTATC<br>CTATAGCGCAGAGCACCTGGCACGTACCATAAGACAGATGG<br>TGATCAACCCCGAAGAAGTGATCATCCCAGATGCAGCTACC<br>CAGGAATCACTCCACACAAAGCTGAAG
IL8L (*CXCLi2*)	HaMeCXCLi2-S1	150	CCGGTGCCAGTGCATAGCCACTCATTCCCGGTTCATTCCCC<br>CGAAATCCATCCAGGATGTGAAGCTGACACAGAGCGGCCCC<br>CACTGCAAGAACGTTGAAGTCATAGCTACTCTGAAGAATGG<br>CAAAGAGGTGTGCTTGGAGCCCACTGC
IL10	HaMeIL10-S1	150	CAGCGCAGCATGAGCGACCTGGGCAACCTCCTGCTGAACCT<br>GAGAGCCACGATGAGACTCTGTCACAAATTCTTCACCTGTG<br>AGGAGAGGAGCAGGAGCATGGAGCACATCAAGGAGACCTTC<br>AGCAGGATGAGCAGGAATGGAATCTAC
IL18	HaMeIL18-S1	150	CCATGCCCAGAGCTGGGGTGCCCGTGGCCTTCAGTGTCCAG<br>GTGGAAAACAAGAGTTATTACATGTGCTGTGAGAGGGAATG<br>TGGAGAAATGATCGTGAGGTTCAAGGAAGGAGAGGTTCCCA<br>AAGAAATTCCTGGTGAAAGCAACATCC
TGFB2	HaMeTGFB2-S1	150	GCTGTTCATGAATGGCTCCATCACAGAGACAGGAATCTTGG<br>ATTTAAGATAAGCTTACATTGTCCATGCTGTACCTTCGTAC<br>CTTCCAATAATTACATCATCCCAAATAAAAGTGAGGAGCTT<br>GAAGCAAGATTTGCAGGTATTGATGAC
TNFSF15	HaMeTNFSF15-S1	150	GAGAGGGGCTCTCACTTTCTGAAGCAACGAGCAGTGGCTGC<br>TGTTACAGACACACTTTCCAGTGCAGAGAAGCCACGAGCTC<br>ACCTGACAGTGAAGAGGCAGGACCTGGCCAGCGCCGTGGGG<br>AGCCACATGCCCATCCTGCAGTGGGAG

1.4. Selection of a reference gene for the RT-qPCR assays

The choice of an appropriate reference gene was done based on the GeNorm analysis (Vandesompele et al., 2002) evaluating gene expression stability in 20 equimolar samples representing five tissues of four individuals. From the two genes (*28SrRNA* and *GAPDH*) identified by GeNorm as the best reference combination, RefFinder (Xie et al., 2012) selected *28SrRNA* as the most stably expressed reference. To further support our reference gene selection, we compared the results for *IL1B* and *IL6* expression across our selection of tissues for data normalised independently on *28SrRNA* and *GAPDH* (stQ, see below). This analysis showed high correlation between both sets of results (for both genes $r > 0.87$, $P \ll 0.001$). Therefore, *28SrRNA* was used as a single universal reference gene in all further RT-qPCR assays in this study.

2 Results

2.1. Tissue-specific variation in cytokine expression response to MG

Fig. S1. Selection of candidate tissues based on tissue-specific variation in cytokine expression at day post-inoculation 6 (DPI 6) between control individuals (n = 2) and individuals inoculated with the MG isolate NC2006 (n = 2). The differences in mean normalised gene expression between treatment and control individuals was calculated using the relative quantification method (Pfaffl, 2001) based on the relative expression ratio R: $R = (E_T)^{\Delta CqT}/(E_R)^{\Delta CqR}$, where E_T is the mean amplification efficiency of the particular assay for a target gene (cytokine), E_R is the mean amplification efficiency of the particular assay for a reference gene (*28SrRNA*), the ΔCqT is the difference in Cq values between control mean and the treatment mean in the target gene (cytokine) and ΔCqR is the difference in Cq values between control mean and the treatment mean in the reference gene (*28SrRNA*). This calculation was done for two selected genes (*IL1B* and *TGFB2*) across the whole panel of 15 tissues. Red arrows indicate tissues selected for further research, asterisks indicate tissues with samples of low RIN values (liver RIN = 5.2, trachea RIN = 3.4 – these tissue types were excluded from further comparisons).

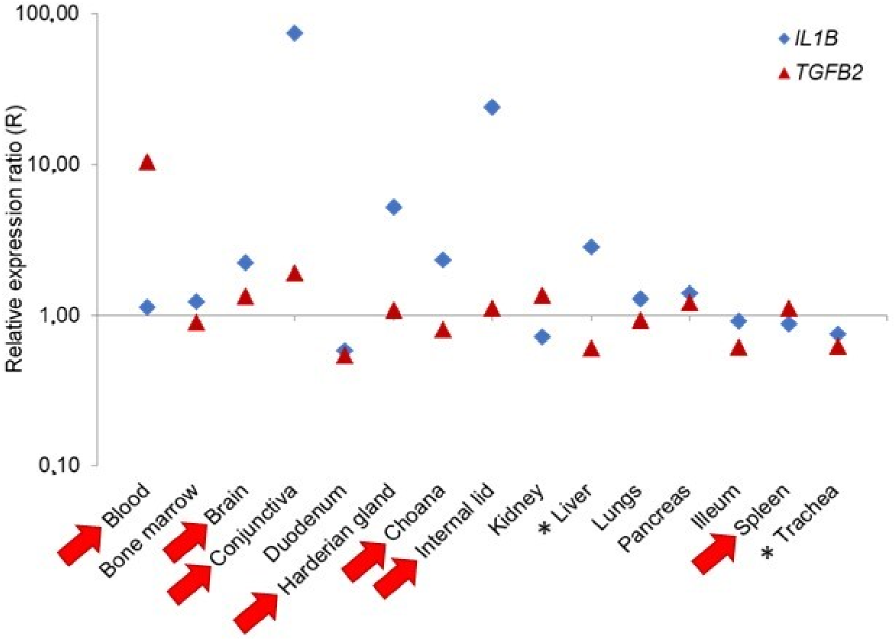

Table S5. Relative differential cytokine expression across the seven selected tissues at post-inoculation day 6 (DPI 6) investigated in four control individuals and four individuals inoculated with the MG isolate NC2006. Standardised quantity (stQ) was calculated as $Q_{TARGET}/Q_{28SrRNA}$, where relative mRNA quantity Q is obtained by equation $Q = E^{\Delta Cq}$, where E is the amplification efficiency, and ΔCq is the difference between the lowest Cq value for the gene in the data set and the sample Cq. *28SrRNA* was used as a reference. Wilcoxon test results are shown. Tissues: Bl = blood, Br = brain, Cj = conjunctiva, HG = Harderian gland, Ch = upper respiratory tract and choana, iL = internal eye lid, Sp = spleen.

Tissue	*IL1B* stQ	*IL6* stQ	*IL10* stQ	*IL18* stQ	*TGFB2* stQ	*CXCLi2* stQ	*TNFSF15* stQ
Bl							W = 1<br>P = 0.057
Br	W = 0<br>P = 0.029					W = 1<br>P = 0.052	
Cj	W = 0<br>P = 0.029	W = 0<br>P = 0.029	W = 0<br>P = 0.029			W = 0<br>P = 0.029	W = 0<br>P = 0.029
HG	W = 0<br>P = 0.029		W = 0<br>P = 0.029				
Ch			W = 1<br>P = 0.057			W = 1<br>P = 0.057	
iL	W = 0<br>P = 0.029	W = 0<br>P = 0.029	W = 0<br>P = 0.029			W = 0<br>P = 0.029	W = 0<br>P = 0.029
Sp							

Table S6. Absolute differential cytokine expression across the seven selected tissues at post-inoculation day 6 (DPI 6) investigated in four control individuals and four individuals inoculated with the MG isolate NC2006. Absolute quantity (A) was calculated as target copy number per ng of total extracted RNA. Wilcoxon test results are shown. Tissues: Bl = blood, Br = brain, Cj = conjunctiva, HG = Harderian gland, Ch = upper respiratory tract and choana, iL = internal eye lid, Sp = spleen.

Tissue	*IL1B* A	*IL6* A	*IL10* A	*IL18* A	*TGFB2* A	*CXCLi2* A	*TNFSF15* A
Bl	W = 1<br>P = 0.057			W = 0<br>P = 0.029			W = 0<br>P = 0.029
Br	W = 0<br>P = 0.029						
Cj	W = 0<br>P = 0.029	W = 0<br>P = 0.029	W = 0<br>P = 0.029	W = 1<br>P = 0.057		W = 0<br>P = 0.029	W = 0<br>P = 0.029
HG	W = 0<br>P = 0.029		W = 0<br>P = 0.029				W = 1<br>P = 0.057
Ch			W = 1<br>P = 0.057			W = 1<br>P = 0.057	
iL	W = 0<br>P = 0.029	W = 0<br>P = 0.029	W = 0<br>P = 0.029			W = 0<br>P = 0.029	W = 0<br>P = 0.029
Sp							

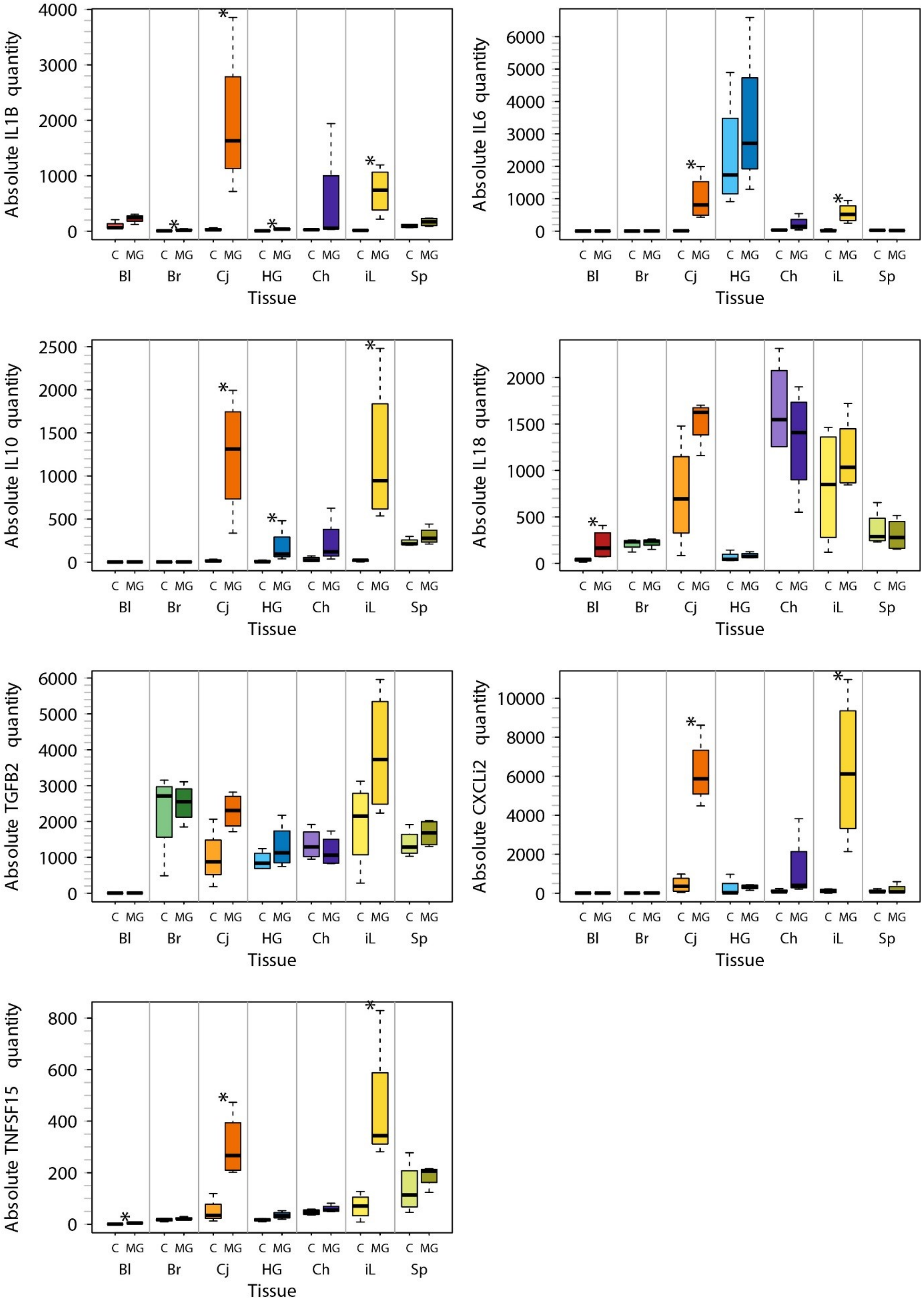

Fig. S2. Tissue-specific absolute differential cytokine gene expression in NC2006 infected house finches and controls on day post-inoculation 6 (DPI6). The boxplots indicating median with the line, upper and lower quartile with the box range and data range with the bars

is based on the absolute quantity (A, transcript copy number per ng of RNA). Tissue types are shown on the x axis highlighted with colour: red – blood (Bl), green – brain (Br), orange – conjunctiva (Cj), blue – Harderian gland (HG), purple – choana and upper respiratory tract (Ch), yellow – nictitating membrane = internal eyelid (iL), light green – spleen (SP). Treatment type: C = control (light colours), MG = inoculation with MG isolate NC2006 (dark colours). Asterisks indicate significant difference in gene expression in the tissue (Wilcoxon test, $p < 0.050$). The cytokine absolute expression data show significant differential expression of *IL1B* in brain, conjunctiva, Harderian gland and internal lid, of *IL6* in conjunctiva and internal lid, of *IL10* in conjunctiva, Harderian gland and internal lid, of *IL18* in blood, of *CXCLi2* in conjunctiva and internal lid, and of *TNFSF15* in blood, conjunctiva and internal lid (Wilcoxon test, in all cases $W = 0$, $p = 0.029$; given the small sample size used for this pilot exploration analysis, these results must be taken with some caution).

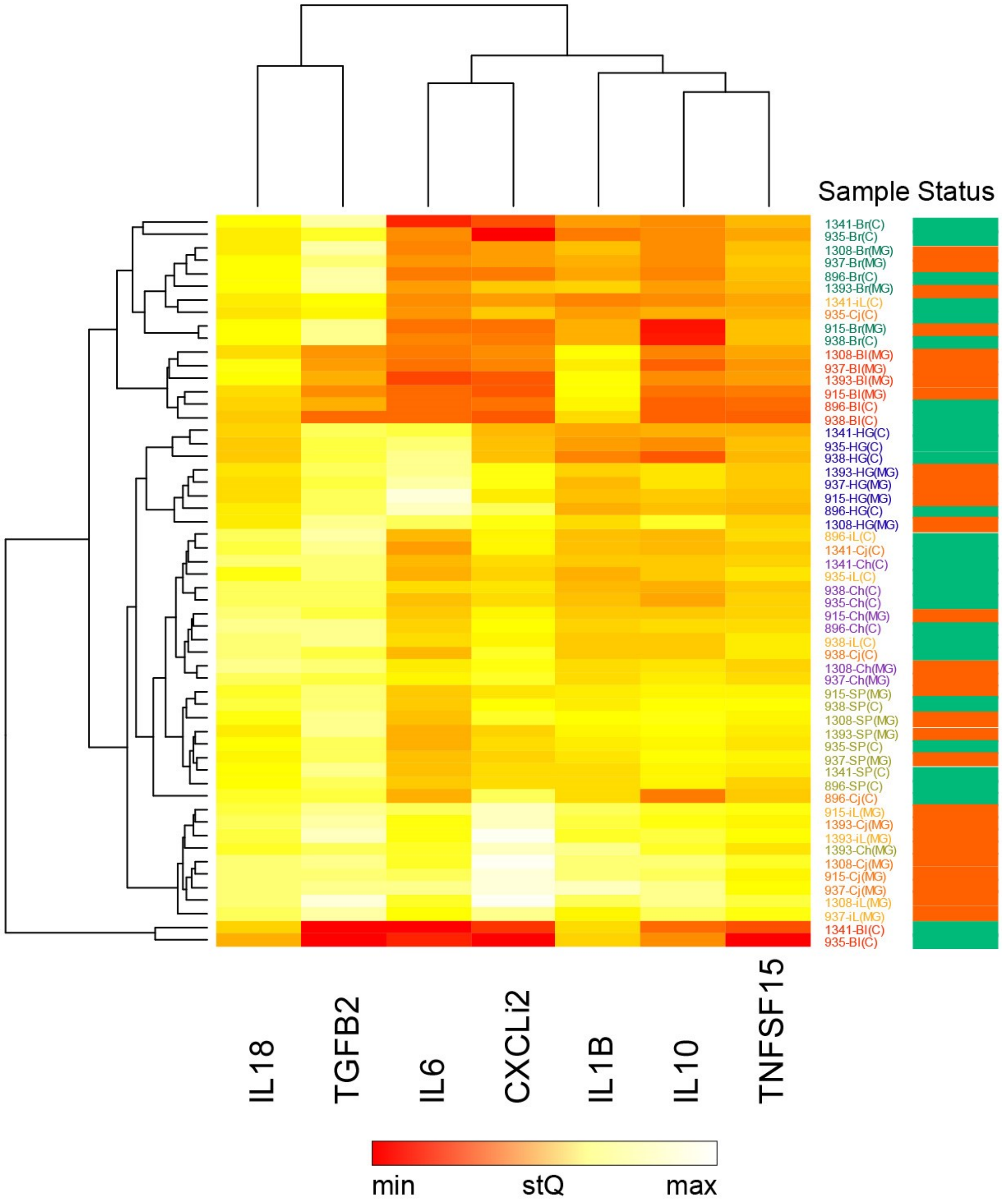

Fig. S3. Heatmap of differential cytokine gene expression in NC2006 infected house finches and controls on day post-inoculation 6 (DPI6). The heatmap is based on the absolute quantity (A, transcript copy number per ng of RNA). Gene names are shown at the bottom of the chart with their codes. Low gene expression is indicated in red, high gene expression in white. Tissue type is highlighted with colour of the sample label: dark red – blood (Bl), green – brain (Br), light red – conjunctiva (Cj), purple – choana and upper respiratory tract (Ch), dark blue – Harderian gland (HG), light blue – spleen (SP), orange – internal eye lid (iL). Dendrograms showing the clustering of the cytokine expression patterns were constructed using UPGMA method.

Table S7. Correlations in relative cytokine expression across tissues. Log_2-transformed data were used as input for the analysis. Above diagonal are shown the Pearson's correlation coefficients, below diagonal are given the Holm's adjusted p-values. Component loadings to the PC1 to PC3 scores calculated by the Principal component analysis are provided in the last three columns.

	CXCLi2	*IL1B*	*IL6*	*IL10*	*IL18*	*TGFB2*	*TNFSF15*	PC1	PC2	PC3
CXCLi2	1	0.383	0.772	0.761	0.460	0.541	0.787	-0.492	-0.034	-0.151
IL1B	0.029	1	0.063	0.535	0.447	-0.298	0.252	-0.215	0.669	-0.198
IL6	<<0.001	1.000	1	0.527	-0.029	0.442	0.478	-0.353	-0.306	-0.547
IL10	<<0.001	<0.001	<0.001	1	0.378	0.276	0.676	-0.431	0.193	-0.251
IL18	<0.001	0.006	1.000	0.029	1	0.211	0.536	-0.288	0.372	0.606
TGFB2	<0.001	0.154	0.006	0.207	0.356	1	0.723	-0.318	-0.523	0.366
TNFSF15	<<0.001	0.243	0.003	<<0.001	<0.001	<<0.001	1	-0.466	-0.093	0.273

2.2. Temporal dynamics of the cytokine expression response to MG

Table S8. Minimum adequate models (MAMs) of the temporal dynamics in *IL1B* and *IL10* expression in response to MG-NC2006 infection in internal eyelid and Harderian gland, based on relative quantification data.

MAM / variable	Df	F	P
Log_2R(IL1B) ~ Tissue + DPI	3/44	24.18	<<0.001
Tissue	1/44	61.85	<<0.001
DPI	2/44	5.35	0.008
Log_2R(IL10) ~ Tissue + DPI + Tissue:DPI	5/42	14.35	<<0.001
Tissue	3/42	9.74	<<0.001
DPI	4/42	13.54	<<0.001
Tissue:DPI	2/42	5.82	0.006

Table S9. Minimum adequate models (MAMs) of the temporal dynamics in *IL1B* and *IL10* expression in response to MG-NC2006 infection in internal eyelid and Harderian gland, based on absolute quantification data.

MAM / variable	Df	F	P
Log_2A(IL1B) ~ Tissue + DPI	3/44	17.81	<<0.001
Tissue	1/44	48.02	<<0.001
DPI	2/44	2.70	0.078
Log_2A(IL10) ~ Tissue + DPI + Tissue:DPI	5/42	12.1	<<0.001
Tissue	3/42	11.04	<<0.001
DPI	4/42	8.73	<<0.001
Tissue:DPI	2/42	3.76	0.031

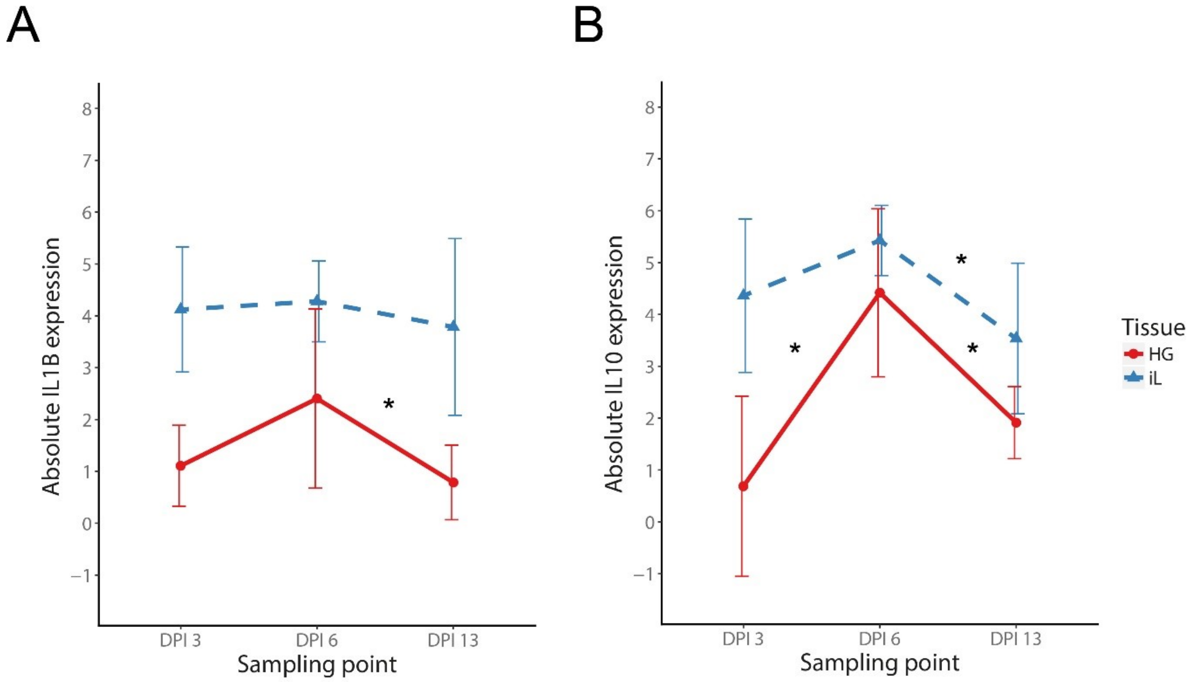

Fig. S4. **Temporal dynamics of *IL1B* (A) and *IL10* (B) absolute expression in response to MG-NC2006 infection in selected tissues across three different time points, based on absolute quantification (A).** Mean ± standard deviation is shown. Red circles represent values in Harderian gland, blue triangles indicate values in internal eyelid. DPI = day post-inoculation. Asterisks mark significant differences between DPI in the respective tissues (Tukey's post-hoc test $P_{adj} < 0.050$). Although in both genes there is apparent peak of the response 6 days post infection (DPI 6) in both tissues, Tukey's post-hoc test showed significant differences between the sampling time points in internal eyelid only in *IL10* (DPI 6-13: P = 0.027), while in HG the DPI 6 was significantly different from the other DPI in both genes (*IL1B*: DPI 6-13: P = 0.044; *IL10*: DPI 6-3: P < 0.001, DPI 6-13: P = 0.010).

2.3. Differences between cytokine expression responses to original and evolved MG isolate

Table S10. Minimum adequate models (MAMs) of variation between infection with *Mycoplasma gallisepticum* isolates VA1994 and NC2006 in triggering *IL1B*, *IL6*, *IL10*, *CXCLi2* and *TNFSF15* expression in internal eyelid (nictitating membrane) across three different time points. Based on the absolute expression ratio (A) data.

MAM / variable	Df	F	P
$Log_2A(IL1B) \sim Treatment$	1/46	18.68	<<0.001
$Log_2A(IL6) \sim Treatment$	1/46	6.75	0.013
$Log_2A(IL10) \sim Treatment + DPI$	3/44	7.51	<0.001
Treatment	1/44	9.35	0.004
DPI	2/44	6.59	0.003
$Log_2A(CXCLi2) \sim Treatment + DPI$	3/44	7.44	<0.001
Treatment	1/44	16.29	<0.001
DPI	2/44	3.02	0.059
$Log_2A(TNFSF15) \sim Treatment + DPI$	5/42	4.55	0.002
Treatment	3/42	6.88	<0.001
DPI	4/42	1.83	0.141
Treatment:DPI	2/42	3.65	0.035

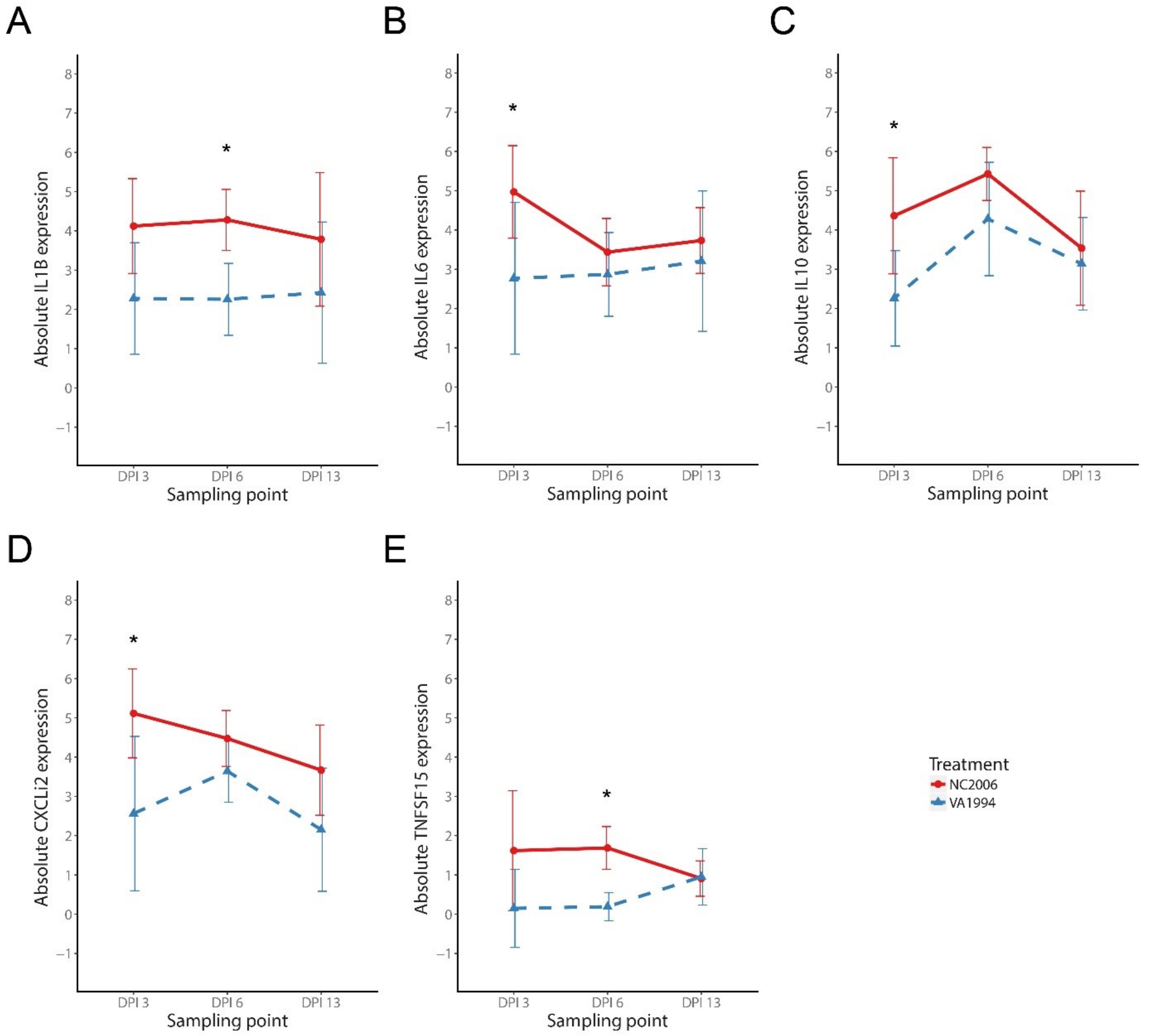

Fig. S5. Effects of experimental infection with *Mycoplasma gallisepticum* isolates VA1994 and NC2006 on absolute *IL1B* (A), *IL6* (B), *IL10* (C), *CXCLi2* (D) and *TNFSF15* (E) expression in internal eyelid (nictitating membrane) across three different time points. The data are shown as absolute expression ratio (A) mean ± SD relative expression quantities. Blue triangles indicate VA1994 isolate data, red circles represent NC2006 isolate data. Asterisks indicate significant difference in gene expression in the tissue (Tukey's post-hoc test, $P < 0.050$). Sampling points: DPI 3 = 3 days post infection, DPI 6 = 6 days post infection, DPI 13 = 13 days post infection. Tukey's post-hoc test showed significant differences between the MG isolates in internal eyelid in *IL1B* (DPI 6: $P < 0.001$), *IL6* (DPI 3: $P = 0.022$), *IL10* (DPI 3: $P = 0.012$), *CXCLi2* (DPI 3: $P = 0.010$), *TNFSF15* (DPI 6: $P << 0.001$). No significant difference was found in IL18 and TGFB2 ($P > 0.050$).

Table S11. Minimum adequate models (MAMs) of variation between infection with *Mycoplasma gallisepticum* isolates VA1994 and NC2006 in triggering *IL1B* and *IL10* expression in Harderian gland across three different time points. Based on the absolute expression ratio (A) data.

MAM / variable	Df	F	P
$Log_2A(IL1B)$ ~ Treatment + DPI	3/44	6.59	<0.001
Treatment	1/44	7.00	0.011
DPI	2/44	6.39	0.004
$Log_2A(IL10)$ ~ DPI	2/45	16.07	<<0.001

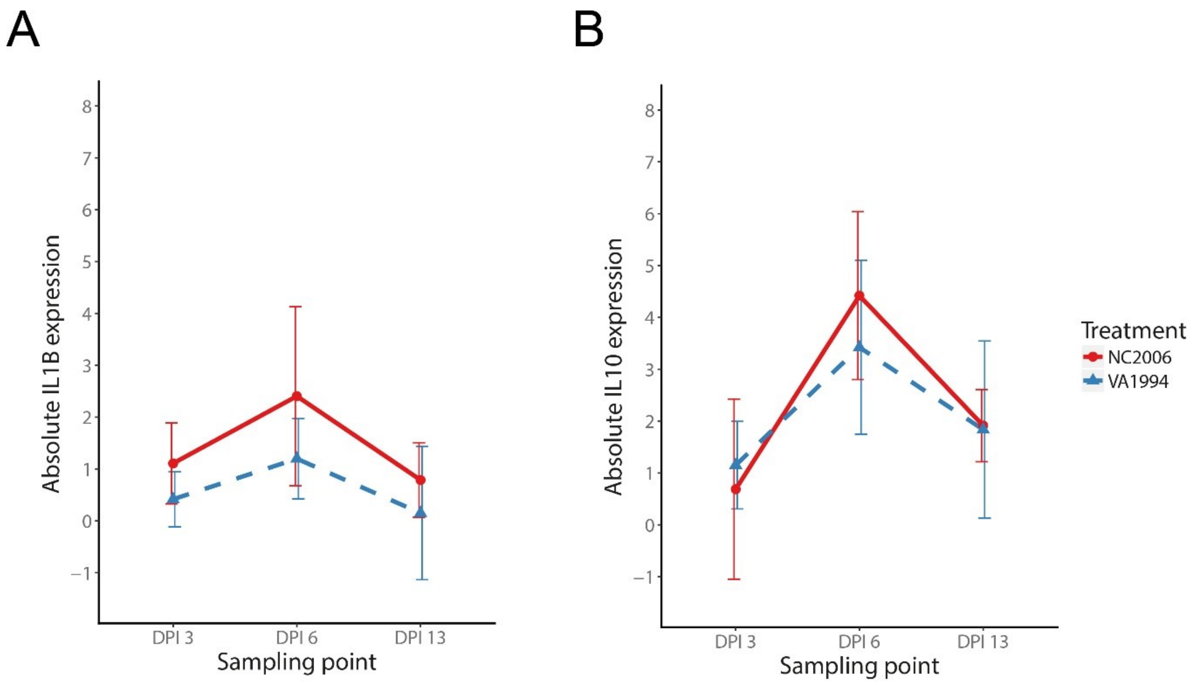

Fig. S6. Effects of experimental infection with *Mycoplasma gallisepticum* isolates VA1994 and NC2006 on *IL1B* (A) and *IL10* (B) expression in Harderian gland across three different time points. The data are shown as absolute expression ratio (A) mean ± SD relative expression quantities. Blue triangles indicate VA1994 isolate data, red circles represent NC2006 isolate data. Tukey's post-hoc test showed no significant differences between the MG isolates in gene expression in the tissue (in all cases P > 0.050). Sampling points: DPI 3 = 3 days post infection, DPI 6 = 6 days post infection, DPI 13 = 13 days post infection.

Table S12. Minimum adequate models (MAMs) for effects of infection with *Mycoplasma gallisepticum* isolates VA1994 and NC2006 on house finch expression of cytokines *IL1B* and *IL10* across three different tissues at day post infection 6 (DPI 6). Based on the relative expression ratio (R) data (i.e. *28SrRNA*- and control-normalised relative expression quantities). Treatment = inoculation with VA1994 or NC2006; Tissue = upper respiratory tract, Harderian gland or internal eyelid (nictitating membrane).

MAM / variable	Df	Chi	P
$Log_2R(IL1B)$ ~ Treatment + Tissue + Treatment:Tissue	5/3	22.56	<0.001
Treatment	3/5	11.73	0.008
Tissue	4/4	20.98	<0.001
Treatment:Tissue	2/6	10.14	0.006
$Log_2R(IL10)$ ~ Treatment + Tissue + Treatment:Tissue	5/3	26.88	<<0.001
Treatment	3/5	6.88	0.076
Tissue	4/4	26.26	<<0.001
Treatment:Tissue	2/6	6.25	0.044

3 References

Pfaffl, M.W., 2001. A new mathematical model for relative quantification in real-time RT-PCR. Nucleic Acids Res. 29. https://doi.org/10.1093/nar/29.9.e45

Vandesompele, J., De Preter, K., Pattyn, F., Poppe, B., Van Roy, N., De Paepe, A., Speleman, F., 2002. Accurate normalization of real-time quantitative RT-PCR data by geometric averaging of multiple internal control genes. Genome Biol. 3. https://doi.org/10.1186/gb-2002-3-7-research0034

Xie, F., Xiao, P., Chen, D., Xu, L., Zhang, B., 2012. miRDeepFinder: a miRNA analysis tool for deep sequencing of plant small RNAs. Plant Mol. Biol. https://doi.org/10.1007/s11103-012-9885-2
